# Physiological and emotional assessment of college students using wearable and mobile devices during the 2020 COVID-19 lockdown: An intensive, longitudinal dataset

**DOI:** 10.1016/j.dib.2024.110228

**Published:** 2024-03-04

**Authors:** Sina Labbaf, Mahyar Abbasian, Brenda Nguyen, Matthew Lucero, Maryam Sabah Ahmed, Asal Yunusova, Alexander Rivera, Ramesh Jain, Jessica L. Borelli, Nikil Dutt, Amir M. Rahmani

**Affiliations:** a Department of Computer Science, University of California, Irvine, CA 92697, USA; b Department of Social Ecology, University of California, Irvine, CA 92697, USA; c Institute of Future Health, University of California, Irvine, CA 92697, USA; d School of Nursing, University of California, Irvine, CA 92697, USA

**Keywords:** Raw wearable physiological signals, Photoplethysmography (PPG) waveforms, Heart rate and heart rate variability, Self-reported real-life mental health data, Longitudinal sleep and physical activity, Ecological momentary assessments (EMAs), Inertial measurement unit waveform, Personal responses to 2020 events

## Abstract

This dataset was collected from university students before, during, and after the COVID-19 lockdown in Southern California. Data collection happened continuously for the average of 7.8 months (*SD*=3.8, *MIN*=1.0, *MAX*=13.4) from a population of 21 students of which 12 have also completed an exit survey, and 7 started before the California COVID-19 lockdown order. This multimodal dataset included different means of data collection such as Samsung Galaxy Watch, Oura Ring, a Life-logger app named Personicle, a questionnaire mobile app named Personicle Questions, and periodical and personalised surveys. The dataset contains raw data from Photoplethysmogram (PPG), Inertial measurement unit (IMU), and pressure sensors in addition to processed data on heart rate, heart rate variability, sleep (bedtime, sleep stages, quality), and physical activity (step, active calories, type of activity). Ecological momentary assessments were collected from participants on daily and weekly bases containing their Positive and Negative Affect Schedule (PANAS) questionnaire and their emotional responses to COVID-19 and their health. Subjective data was also collected through monthly surveys containing standard mood and mental health surveys such as Beck Depression Inventory II (BDI-II), Brief Symptom Inventory (BSI), GAD-7, Inclusion of Other in the Self Scale (IOS-Partner), Acceptability of Intervention Measure (AIM), Intervention Appropriateness Measure (IAM), Feasibility of Intervention Measure (FIM), Experiences in Close Relationships Scale Short Form (ECR-S), UCLA Three-Item Loneliness Scale (ULS), Multidimensional Scale of Perceived Social Support (MSPSS), Investment Model Scale (IMS), Conflict Management Scale (CMS), etc in addition to their response to important events and COVID-19. This dataset can be used to study emotions, mood, physical activity, and lifestyle of young adults through longitudinal subjective and objective measures. This dataset also contains valuable data regarding adjustment of lifestyle and emotions during the events of 2020 and 2021 including COVID-19 discovery and lockdown, Black Life Matter movement, 2020 US presidential elections, etc. On average, participants engaged in the EMA collection study at a rate of 86% (SD=10, MIN=65, MAX=99). Smartwatch usage saw an average participation rate of 51% (SD=20, MIN=16, MAX=88), while engagement with the Oura ring averaged at 85% (SD=12, MIN=60, MAX=99).

Specifications Table

**Table:** 

Subject	Psychology
Specific subject area	mobile health monitoring
Data format	Raw, Analyzed, Questionnaires
Type of data	Table
Data collection	The data are collected from wearable wristband smartwatch (Samsung Galaxy Gear Sport), smart ring (Oura Ring), Android questionnaire mobile application (Personicle Questions), Android life-logging mobile application (Personicle), Questionnaire surveys (Qualtrics). The data is collected from full-time students from the University of California, Irvine aged 18-22 years, who own an Android smartphone as their primary phone that is compatible with study devices and applications. Participants are ineligible if they are parents, married, returning to school after a period of ≥3 years, or unable to speak/write English fluently.
Data source location	Institution: University of California, Irvine City: Irvine State: California Country: United States of America
Data accessibility	Repository name: Dryad Data identification number: 10.7280/D1WH6T Direct URL to data: https://doi.org/10.7280/D1WH6T[2]
Related research article	[1] A. Yunusova, J. Lai, A.P. Rivera, S. Hu, S. Labbaf, A.M. Rahmani, N. Dutt, R.C. Jain, J.L. Borelli, 2021. Assessing the mental health of emerging adults through a mental health app: Protocol for a prospective pilot study, JMIR Res. Protoc. 10 e25775. https://doi.org/10.2196/25775.

## 1 Value of the Data

• Provides longitudinal raw data in everyday settings capturing everyday activities and lifestyle
• Covering different dimensions of participants lifestyle such as sleep, activity, emotions, mental health, physical health, and personal events
• It is a unique dataset that contains data from before, during, and after COVID-19 lockdown, black lives matter, 2020 presidential election, and other events of 2020 and 2021 from the same people

## 2 Data Description

### 2.1 Population information

Table 1 shows the demographic distribution of the participants’ population. To preserve the privacy of the participants the demographic data of each participant will only be available upon request.

**Table 1:** Demographics of the study population.

Demographic	Distribution	Number
Ethnicity	Asian	13
	Hispanic or Latino	4
	White	3
	Other	1
Gender	Female	14
	Male	6
	Other	1
Birth year	1999 and before	6
	2000	7
	2001 and after	8
College start year	2016 and before	5
	2017	9
	2018 and after	8

### 2.2 Participant's data

At the root of the dataset directory tree each participant has their own folder. These folders are names from “par_1” to “par_21” representing each of the participant ids. Each participant's folder contains all the data collected from that participant in its subfolders. In each participant's folder there are four subfolders for different modalities of the study. These modalities are a) EMAs: These are the questionnaires that were collected throughout the study by the Personicle Questionnaire mobile application. b) Oura: These are the data collected from Oura ring. c) Samsung: Samsung data were collected from Samsung Galaxy Gear Sport smartwatch. d) Assessments: These data were collected by the study coordinators 6 times throughout the study asking participants about their demographic information, standard questionnaires, and their participation in the study. Also another final questionnaire about their emotional responses toward important events of 2019 and 2020 and personal events in their life during their participation in the study. e) Personicle: Lifelogging and location data collected by Personicle Android app. Table 2 shows the different modalities of these subfolders.

**Table 2:** Dataset subfolders organization.

Subfolder name	Collected by	Description
ema	Personicle Questionnaires App	This folder contains all the responses to weekly and daily ecological momentary assessments (EMAs) sent to participants' mobile phones. Participants were notified and reminded to fill these out by mobile push notifications.
oura	Oura ring v1	This folder contains all the sensor recordings from participants' Oura ring. The ring was used to monitor sleep and activity.
samsung	Samsung Galaxy Gear Sport watch	This folder contains all the data collected by Samsung Gear sport smartwatch. This folder provides some raw sensor reading from the watch as well as some processed information.
assessment	Qualtrics	This folder contains all the responses to the assessments captured within Qualtrics. These assessments were taken at 0 weeks, 4 weeks, and 16 weeks of the study.
personicle	Personicle Mobile App	This folder contains data from Personicle mobile application. Personicle was used to collect life-logging and behavioural data from participants.

### 2.3 Dataset subfolders

#### 2.3.1 EMA

EMA data folders contain two comma separated vector files “daily.csv” and “weekly.csv”. “daily.csv” represents the questionnaires that participants were supposed to respond to everyday. and “weekly.csv” is the questionnaire they responded to at the end of each week on Sundays. These EMAs were collected by Personicle Questions mobile application. The content of each of these files are described in Tables 3 and 4.

**Table 3:** Ema/daily.csv.

Column name	Description	Range	Type of variable
submission_timestamp	Epoch timestamp of the submission time of the survey	-	Timestamp (milliseconds)
start_timestamp	Epoch timestamp of the time that participant started to fill out the survey	-	Timestamp (milliseconds)
Active	Answer to “Indicate the extent you have felt this way today: Active” Indicated by: • Very slightly • A little • Moderately • Quite a bit • Extremely	0 - 100	Number
Afraid	Answer to “Indicate the extent you have felt this way today: Afraid” Indicated by: • Very slightly • A little • Moderately • Quite a bit • Extremely	0-100	Number
Alert	Answer to “Indicate the extent you have felt this way today: Alert” Indicated by: • Very slightly • A little • Moderately • Quite a bit • Extremely	0-100	Number
Ashamed	Answer to “Indicate the extent you have felt this way today: Ashamed” Indicated by: • Very slightly • A little • Moderately • Quite a bit • Extremely	0-100	Number
Attentive	Answer to “Indicate the extent you have felt this way today: Attentive” Indicated by: • Very slightly • A little • Moderately • Quite a bit • Extremely	0-100	Number
Determined	Answer to “Indicate the extent you have felt this way today: Determined” Indicated by: • Very slightly • A little • Moderately • Quite a bit • Extremely	0-100	Number
Distressed	Answer to “Indicate the extent you have felt this way today: Distressed” Indicated by: • Very slightly • A little • Moderately • Quite a bit • Extremely	0-100	Number
Enthusiastic	Answer to “Indicate the extent you have felt this way today: Enthusiastic” Indicated by: • Very slightly • A little • Moderately • Quite a bit • Extremely	0-100	Number
Excited	Answer to “Indicate the extent you have felt this way today: Enthusiastic” Indicated by: • Very slightly • A little • Moderately • Quite a bit • Extremely	0-100	Number
Guilty	Answer to “Indicate the extent you have felt this way today: Guilty” Indicated by: • Very slightly • A little • Moderately • Quite a bit • Extremely	0-100	Number
Hostile	Answer to “Indicate the extent you have felt this way today: Hostile” Indicated by: • Very slightly • A little • Moderately • Quite a bit • Extremely	0-100	Number
Inspired	Answer to “Indicate the extent you have felt this way today: Inspired” Indicated by: • Very slightly • A little • Moderately • Quite a bit • Extremely	0-100	Number (Slider)
Interested	Answer to “Indicate the extent you have felt this way today: Interested” Indicated by: • Very slightly • A little • Moderately • Quite a bit • Extremely	0-100	Number (Slider)
Irritable	Answer to “Indicate the extent you have felt this way today: Irritable” Indicated by: • Very slightly • A little • Moderately • Quite a bit • Extremely	0-100	Number (Slider)
Jittery	Answer to “Indicate the extent you have felt this way today: Jittery” Indicated by: • Very slightly • A little • Moderately • Quite a bit • Extremely	0-100	Number (Slider)
Nervous	Answer to “Indicate the extent you have felt this way today: Nervous” Indicated by: • Very slightly • A little • Moderately • Quite a bit • Extremely	0-100	Number (Slider)
Proud	Answer to “Indicate the extent you have felt this way today: Proud” Indicated by: • Very slightly • A little • Moderately • Quite a bit • Extremely	0-100	Number (Slider)
Scared	Answer to “Indicate the extent you have felt this way today: Scared” Indicated by: • Very slightly • A little • Moderately • Quite a bit • Extremely	0-100	Number (Slider)
Strong	Answer to “Indicate the extent you have felt this way today: Strong” Indicated by: • Very slightly • A little • Moderately • Quite a bit • Extremely	0-100	Number (Slider)
Upset	Answer to “Indicate the extent you have felt this way today: Upset” Indicated by: • Very slightly • A little • Moderately • Quite a bit • Extremely	0-100	Number (Slider)
Covid_worried	Answer to “How worried were you about contracting COVID-19 today?” Indicated by: • Not worried at all • Extremely worried	0-100	Number (Slider)
daily_feeling	Answer to “Can you please take a few minutes to tell us in greater depth how you are feeling today, and what contributed to how you're feeling?” **This field is only available upon request to protect participants’ privacy.**	-	Text
health_worried	Answer to “Please rate how worried you felt about your health today?” Indicated by: • Not worried at all • Extremely worried	0-100	Number (Slider)
*_last_modified	Epoch timestamp of the last time that the participant has modified the value of this component in their app. If “undefined” it means that the participant didn't interact with this component.	-	Timestamp (milliseconds)

daily.csv content.

**Table 4:** Ema/weekly.csv.

Column name	Description	Range	Type of variable
submission_timestamp	Epoch timestamp of the submission time of the survey	-	Timestamp (milliseconds)
start_timestamp	Epoch timestamp of the time that participant started to fill out the survey	-	Timestamp (milliseconds)
Active	Answer to “Indicate the extent you have felt this way today: Active” Indicated by: • Very slightly • A little • Moderately • Quite a bit • Extremely	0 - 100	Number
Afraid	Answer to “Indicate the extent you have felt this way today: Afraid” Indicated by: • Very slightly • A little • Moderately • Quite a bit • Extremely	0-100	Number
Alert	Answer to “Indicate the extent you have felt this way today: Alert” Indicated by: • Very slightly • A little • Moderately • Quite a bit • Extremely	0-100	Number
Ashamed	Answer to “Indicate the extent you have felt this way today: Ashamed” Indicated by: • Very slightly • A little • Moderately • Quite a bit • Extremely	0-100	Number
Attentive	Answer to “Indicate the extent you have felt this way today: Attentive” Indicated by: • Very slightly • A little • Moderately • Quite a bit • Extremely	0-100	Number
Determined	Answer to “Indicate the extent you have felt this way today: Determined” Indicated by: • Very slightly • A little • Moderately • Quite a bit • Extremely	0-100	Number
Distressed	Answer to “Indicate the extent you have felt this way today: Distressed” Indicated by: • Very slightly • A little • Moderately • Quite a bit • Extremely	0-100	Number
Enthusiastic	Answer to “Indicate the extent you have felt this way today: Enthusiastic” Indicated by: • Very slightly • A little • Moderately • Quite a bit • Extremely	0-100	Number
Excited	Answer to “Indicate the extent you have felt this way today: Excited” Indicated by: • Very slightly • A little • Moderately • Quite a bit • Extremely	0-100	Number
Guilty	Answer to “Indicate the extent you have felt this way today: Guilty” Indicated by: • Very slightly • A little • Moderately • Quite a bit • Extremely	0-100	Number
Hostile	Answer to “Indicate the extent you have felt this way today: Hostile” Indicated by: • Very slightly • A little • Moderately • Quite a bit • Extremely	0-100	Number
Inspired	Answer to “Indicate the extent you have felt this way today: Inspired” Indicated by: • Very slightly • A little • Moderately • Quite a bit • Extremely	0-100	Number (Slider)
Interested	Answer to “Indicate the extent you have felt this way today: Interested” Indicated by: • Very slightly • A little • Moderately • Quite a bit • Extremely	0-100	Number (Slider)
Irritable	Answer to “Indicate the extent you have felt this way today: Irritable” Indicated by: • Very slightly • A little • Moderately • Quite a bit • Extremely	0-100	Number (Slider)
Jittery	Answer to “Indicate the extent you have felt this way today: Jittery” Indicated by: • Very slightly • A little • Moderately • Quite a bit • Extremely	0-100	Number (Slider)
Nervous	Answer to “Indicate the extent you have felt this way today: Nervous” Indicated by: • Very slightly • A little • Moderately • Quite a bit • Extremely	0-100	Number (Slider)
Proud	Answer to “Indicate the extent you have felt this way today: Proud” Indicated by: • Very slightly • A little • Moderately • Quite a bit • Extremely	0-100	Number (Slider)
Scared	Answer to “Indicate the extent you have felt this way today: Scared” Indicated by: • Very slightly • A little • Moderately • Quite a bit • Extremely	0-100	Number (Slider)
Strong	Answer to “Indicate the extent you have felt this way today: Strong” Indicated by: • Very slightly • A little • Moderately • Quite a bit • Extremely	0-100	Number (Slider)
Upset	Answer to “Indicate the extent you have felt this way today: Upset” Indicated by: • Very slightly • A little • Moderately • Quite a bit • Extremely	0-100	Number (Slider)
week_felt	Answer to “Please rate how you felt about your week.” Indicated by: • 0: Completely Negative • 100: Completely Positive	0-100	Number (Slider)
Covid_worried	Answer to “How worried were you about contracting COVID-19 today?” Indicated by: • 0: Not worried at all • 100: Extremely worried	0-100	Number (Slider)
week_points	Answer to “Please write about your high points and low points this week. Please try to be as detailed as possible.” **This field is only available upon request to protect participants’ privacy.**	-	Text
*_last_modified	Epoch timestamp of the last time that the participant has modified the value of this component in their app. If “undefined” it means that the participant didn't interact with this component.	-	Timestamp (milliseconds)

weekly.csv content.

#### 2.3.2 Oura

This subfolder represents the data collected through Oura ring v1. Oura provides data in three modalities: activity, sleep, and readiness. Activity data is available throughout day and night and shows how active the participants were throughout the day and on average each day represented in Tables 5 and 6. It also contains heart rate and heart rate variability of participants during their sleep presentated by Table 7. The other modality is readiness which is a set of different scores that Oura calculates based on the collected data to show the overall wellbeing of participants during each day described in Table 8. The sleep data is generally only available for night time and represent the quality of sleep and different sleep stages at Tables 9 and 10 The descriptions for these fields have been recited from Oura API V1 documentation [3].

**Table 5:** Oura/activity_level.csv.

Column name	Description	Range	Type
timestamp	Epoch timestamp of this data row	-	Timestamp (milliseconds)
activity_level	The numeric level of activity with: • 0: Non-wear • 1: Rest (MET level below 1.05) • 2: Inactive (MET level between 1.05 and 2) • 3: Low intensity activity (MET level between 2 and age/gender dependent limit) • 4: Medium intensity activity • 5: High intensity activity	0-5	Number
activity_class	The actual level of activity either: • non-wear • rest • inactive • low • medium • high	-	Text

activity_level.csv.

**Table 6:** Oura/activity.csv.

Column name	Description	Range	Type
date	The day for this row of data	-	Text (YYYY-MM-DD)
score	An estimation on how well recent physical activity has matched participant's needs. It is calculated as a weighted average of activity score contributors that represent one aspect of suitability of the activity each. (score = score_stay_active*0.15 + score_move_every_hour*0.1 + score_meet_daily_targets*0.25 + score_training_frequency*0.1 + score_training_volume*0.15 + score_recovery_time*0.25)	1-100 (0=miss)	Number
score_stay_active	An indication of how well the participant has managed to avoid inactivity (sitting or standing still) during the last 24 hours. The more inactivity, the lower contributor value. The contributor value is 100 when inactive time during the past 24 hours is below 5 hours. The contributor value is above 95 when inactive time during the past 24 hours is below 7 hours.	1-100 (0=miss)	Number
score_move_every_hour	An indication of how well the participant has managed to avoid long periods of inactivity (sitting or standing still) during the last 24 hours. The score includes a number of continuous inactive periods of 60 minutes or more (excluding sleeping). The more long inactive periods, the lower the value. The score is 100 when no continuous inactive periods of 60 minutes or more have been registered. The contributor value is above 95 when at most one continuous inactive period of 60 minutes or more has been registered.	1-100 (0=miss)	Number
score_meet_daily_targets	An indication of how often the participant has reached their daily activity target during seven last days (100 = six or seven times, 95 = five times).	1-100 (0=miss)	Number
score_training_frequency	An indication of how regularly the participant has had physical exercise during the last seven days. The value is 100 when the participant has got more than 100 minutes of medium or high intensity activity on at least four days during the past seven days. The score value is 95 when the user has got more than 100 minutes of medium or high intensity activity on at least three days during the past seven days.	1-100 (0=miss)	Number
score_training_volume	An indication of how much physical exercise the participant has got during the last seven days. The value is 100 when thes sum of weekly MET minutes is over 2000. The value is 95 when the sum of weekly MET minutes is over 750. There is a weighting function so that the effect of each day gradually disappears.	1-100 (0=miss)	Number
score_recovery_time	An indication of the recovery time during the last seven days. The value is 100 when: 1. The participant has got at least two recovery days during the past 7 days. 2. No more than two days elapsed after the latest recovery day. The value is 95 when: 1. The participant has got at least one recovery day during the past 7 days. 2. No more than three days elapsed after the latest recovery day. Here a day is considered as a recovery day when the amount of high intensity activity did not exceed 100 MET minutes and the amount of medium intensity activity did not exceed 200 MET minutes. The exact limits will be age and gender dependent.	1-100 (0=miss)	Number
cal_active	Energy consumption caused by the physical activity of the day.	-	Number (kcal)
cal_total	Total energy consumption during the day including Basal Metabolic Rate	-	Number (kcal)
daily_movement	Daily physical activity expressed as the amount of walking in meters that is needed to get the same amount of activity	-	Number (meters)
inactivity_alerts	Number of continuous inactive periods of 60 minutes or more during the day.		Number
steps	Total number of steps registered during the day.		Number
non_wear	Number of minutes during the day when the participant was not wearing the ring. Can be used as a proxy for data accuracy, i.e. how well the measured physical activity represents actual total activity.	0-1440	Number (minutes)
rest	Number of minutes during the day spent resting i.e. sleeping or lying down (average MET level of the minute is below 1.05).	0-1440	Number (minutes)
inactive	Number of inactive minutes (sitting or standing still, average MET level of the minute between 1.05 and 2) during the day.	0-1440	Number (minutes)
low	Number of minutes during the day with low intensity activity (e.g. household work, average MET level of the minute between 2 and age dependent limit).	0-1440	Number (minutes)
medium	Number of minutes during the day with medium intensity activity (e.g. walking). The upper and lower MET level limits for medium intensity activity depend on participant's age and gender.	0-1440	Number (minutes)
high	Number of minutes during the day with high intensity activity (e.g. running). The lower MET level limit for high intensity activity depends on the participant's age and gender.	0-1440	Number (minutes)
average_met	Average MET level during the whole day.	-	Number (MET)
met_min_inactive	Total MET minutes accumulated during inactive minutes of the day.	-	Number (MET)
met_min_low	Total MET minutes accumulated during low intensity activity minutes of the day.	-	Number (MET)
met_min_medium	Total MET minutes accumulated during medium intensity activity minutes of the day.	-	Number (MET)
met_min_high	Total MET minutes accumulated during high intensity activity minutes of the day.	-	Number (MET)
day_start_timestamp	Start of the day that this row of data is representing.	-	Timestamp (milliseconds)
day_end_timestamp	End of the day that this row of data is representing.	-	Timestamp (milliseconds)

activity.csv.

**Table 7:** Oura/heart_rate.csv.

Column name	Description	Range	Type (unit)
timestamp	Epoch timestamp of this data row.	-	Timestamp (milliseconds)
heart_rate	Average heart rate for each 5 minutes of the sleep period.	-	Number (beats/min)
heart_rmssd	The average root mean square of successive heartbeat interval differences for each 5 minutes of the sleep period.	-	Number (milliseconds)

heart_rate.csv.

**Table 8:** Oura/readiness.csv.

Column name	Description	Range	Type (unit)
date	The day for this row of data.	-	Text (YYYY-MM-DD)
score	Oura readiness score.	1-100 (0=miss)	Number
score_activity_balance	Measurement of how the participant's activity level over the past days is affecting their readiness to perform. A 100 indicates that they've been active, but kept from training at their maximum capacity. This has boosted their recovery and helped build up their energy levels.	1-100 (0=miss)	
score_hrv_balance	Indicator of how close the heart rate variability is to the optimum of participant's ideal heart rate variability during their sleep.	1-100 (0=miss)	
score_previous_day	When 100%, it shows a good balance of active time and rest. An exceptionally high amount of inactivity or activity leads to a drop in Readiness Score.	1-100 (0=miss)	
score_previous_night	Oura previous night score.	1-100 (0=miss)	
score_recovery_index	Measures how long it takes for resting heart rate to stabilize during the night. A sign of very good recovery is that resting heart rate stabilizes during the first half of the night, at least 6 hours before wake up time, leaving body time to recover for the next day.	1-100 (0=miss)	
score_resting_hr	Indicator of how close the resting heart rate is to the optimum of participant's ideal resting heart rate during their sleep	1-100 (0=miss)	
score_sleep_balance	Shows if the sleep over the past two weeks is in balance with participant's needs. Typically adults need 7-9 hours of sleep a night to stay healthy, alert, and to perform at their best both mentally and physically.	1-100 (0=miss)	
score_temperature	An indicator of sleep temperature balance. If the temperature rapidly changes during the sleep or shows abnormal values this score will decrease.	1-100 (0=miss)	

readiness.csv.

**Table 9:** Oura/sleep_hypnogram.csv.

Column name	Description	Range	Type
timestamp	Epoch timestamp of this data row.	-	Timestamp
hypnogram_level	The numeric level of sleep hypnogram: • 1 = deep (N3) sleep • 2 = light (N1 or N2) sleep • 3 = REM sleep • 4 = awake	1-4	Number
hypnogram_class	The actual class of sleep either: • deep • light • REM • awake	-	Text

sleep_hypnogram.csv.

**Table 10:** Oura/sleep.csv.

Column name	Description	Range	Type
date	One day prior to the date when the sleep period ended. This is one day before the date that is shown in Oura apps.	**-**	Text (YYYY-MM-DD)
score	Overall sleep quality during the sleep period. It is calculated as a weighted average of sleep score contributors that represent one aspect of sleep quality each. The sleep score contributor values are also available as separate parameters. (scope = 0.1*score_alignment + 0.1*score_deep + 0.15*score_disturbances + 0.1*score_efficiency + 0.1*score_latency + 0.1*score_rem + 0.35*score_total)	1-100	
score_alignment	Circadian alignment's contribution for sleep score. Sleep midpoint time between 12PM and 3AM gives the highest score. The more the midpoint time deviates from that range, the lower the score.	1-100	
score_deep	Represents deep (N3) sleep time's contribution for sleep quality. The value depends on the age of the participant - the younger, the more sleep is needed for good score.	1-100	
score_disturbances	Represents sleep disturbances' contribution for sleep quality. Three separate measurements are used to calculate this contributor value: 1. Wake-up count - the more wake-ups, the lower the score. 2. Got-up count - the more get-ups, the lower the score. 3. Restless sleep (sleep.restless) - the more motion detected during sleep, the lower the score. Each of these three values has weight 0.05 in sleep score calculation.	1-100	
score_efficiency	Represents sleep efficiency's contribution for sleep quality. The higher efficiency, the higher the score.	1-100	
score_latency	Represents sleep onset latency's contribution for sleep quality. A latency of about 15 minutes gives the best score. Latency longer than that may indicate problems falling asleep, whereas a very short latency may be a sign of sleep debt.	1-100	
score_rem	Represents REM sleep time's contribution for sleep quality. The value depends on the age of the user - the younger, the more sleep REM is needed for good score.	1-100	
score_total	Represents total sleep time's contribution for sleep quality. The value depends on the age of the user - the younger, the more sleep is needed for a good score.	1-100	
duration	Total duration of the sleep period.	-	Number (seconds)
awake	Total amount of awake time registered during the sleep period.	-	Number (seconds)
light	Total amount of light (N1 or N2) sleep registered during the sleep period.	-	Number (seconds)
rem	Total amount of REM sleep registered during the sleep period.	-	Number (seconds)
deep	Total amount of deep (N3) sleep registered during the sleep period.	-	Number (seconds)
total	Total amount of sleep registered during the sleep period (total = rem + light + deep).	-	Number (seconds)
onset_latency	Detected latency from getting into bed to the beginning of the first five minutes of persistent sleep.	-	Number (seconds)
midpoint_time	The time in seconds from the start of sleep to the midpoint of sleep. The midpoint ignores awake periods.	-	Number (seconds)
efficiency	Sleep efficiency is the percentage of the sleep period spent asleep (100 * total / duration).	0-100	Number
restless	Restlessness of the sleep time, i.e. percentage of sleep time when the user was moving.	0-100	Number
hr_average	The average heart rate registered during the sleep period.	-	Number (beats / minute)
hr_lowest	The lowest heart rate (5 minutes sliding average) registered during the sleep period.	-	Number (beats / minute)
rmssd	The average HRV calculated with the rMSSD method.	-	Number (milliseconds)
breath_average	Average respiratory rate.	-	Number (breaths per minute)
bedtime_start_midnight_delta	Difference between bedtime start and local midnight.	-	Number (seconds)
bedtime_end_midnight_delta	Difference between bedtime end and local midnight.	-	Number (seconds)
temperature_delta	Skin temperature deviation from the long-term temperature average.	-	Number (Celsius)
bedtime_start_timestamp	Epoch timestamp of the start of the sleep.	-	Timestamp (milliseconds)
bedtime_end_timestamp	Epoch timestamp of the end of the sleep.	-	Timestamp (milliseconds)

sleep.csv.

Metabolic Equivalent (MET) value referred to in this document is a relative measurement of physical activity intensity. The Energy expenditure of a person while resting is 1 and the physical activity intensity of X MET means the person was expending X times more the energy that their resting state.

#### 2.3.3 Samsung

These data were collected using Samsung Galaxy Gear Sport smartwatch. Data files “imu.csv”, “ppg.csv”, and “pressure.csv” are raw data collected using direct sensor reading from the watch. and “pedometer.csv”, and “awake_times.csv” are processed data collected from the watch. All of the data collected directly from Samsung are presented in Table 11, Table 12, Table 13, Table 14, Table 15.

**Table 11:** Samsung/awake_times.csv.

Column name	Description	Unit
timestamp_start	Timestamp indicating the start of an awake period.	Timestamp (milliseconds)
timestamp_end	Timestamp indicating the end of an awake period.	Timestamp (milliseconds)
state	Always “awake”	Text

awake_times.csv.

**Table 12:** Samsung/imu.csv.

Column name	Description	Range	Unit
timestamp	The timestamp of this row of data reading.	-	Timestamp (milliseconds)
accx	Linear acceleration towards the X axis.	-19.6-19.6	Number $\left(m/s^{2}\right)$
accy	Linear acceleration towards the Y axis.	-19.6-19.6	Number $\left(m/s^{2}\right)$
accz	Linear acceleration towards the Z axis.	-19.6-19.6	Number $\left(m/s^{2}\right)$
gyrx	Angular velocity around the X axis.	-573-573	Number $\left({}^{\circ }/s\right)$
gyry	Angular velocity around the Y axis.	-573-573	Number $\left({}^{\circ }/s\right)$
gyrz	Angular velocity around the Z axis.	-573-573	Number $\left({}^{\circ }/s\right)$

imu.csv.

**Table 13:** Samsung/pedometer.csv.

Column name	Description	Unit
timestamp	The timestamp of this row of data reading.	Timestamp (milliseconds)
num_total_steps	Total number of steps since the last reboot of the device to time indicated by timestamp.	Number
num_total_walking_steps	Total number of steps walking since the last reboot of the device to time indicated by timestamp.	Number
num_total_running_steps	Total number of steps running since the last reboot of the device to time indicated by timestamp.	Number
move_distance_meter	Total distance travelled by foot since the last reboot of the device to time indicated by timestamp.	Number (meters)
cal_burn_kcal	Total calories burnt since the last reboot of the device to time indicated by timestamp.	Number (kcal)
last_speed_kmh	Last speed of the device before the timestamp.	Number (km/h)
last_step_freq	Last stepping frequency of the device before timestamp.	Number (steps/second)
last_state_level	Last state of the device before the timestamp between -1 to 2. • -1: unknown state • 0: stop state • 1: walking state • 2: running state	Number
last_state_class	The class of the last_state_level. Either “unknown”, “stop”, “walking”, or “running”.	Text

pedometer.csv.

**Table 14:** Samsung/ppg.csv.

Column name	Description	Range	Unit
timestamp	The timestamp of this row of data reading.	-	Timestamp (milliseconds)
ppg	The heart rate monitor (HRM) LED green sensor measures the amount of green light that is reflected back from a person's blood vessel.	0-4194304	Number
hr	Real-time heart rate extracted from ppg signal.	0-240	Number (beats/minute)

ppg.csv.

**Table 15:** Samsung/pressure.csv.

Column name	Description	Range	Unit
timestamp	The timestamp of this row of data reading	-	Timestamp (milliseconds)
pressure	The atmospheric pressure in the device's surrounding environment.	260-1260	Number (hectopascals)

pressure.csv

In addition to the raw data and processed data collected from the Samsung watch, this subfolder contains two files that are post processed features extracted from the ppg signal by Khatibi et al. [4]. These features are in two files named “hrv_1min.csv” (for one minute time window features) and “hrv_5min.csv” (for 5 minute time window features) presented in Table 16.

**Fig. 1:**
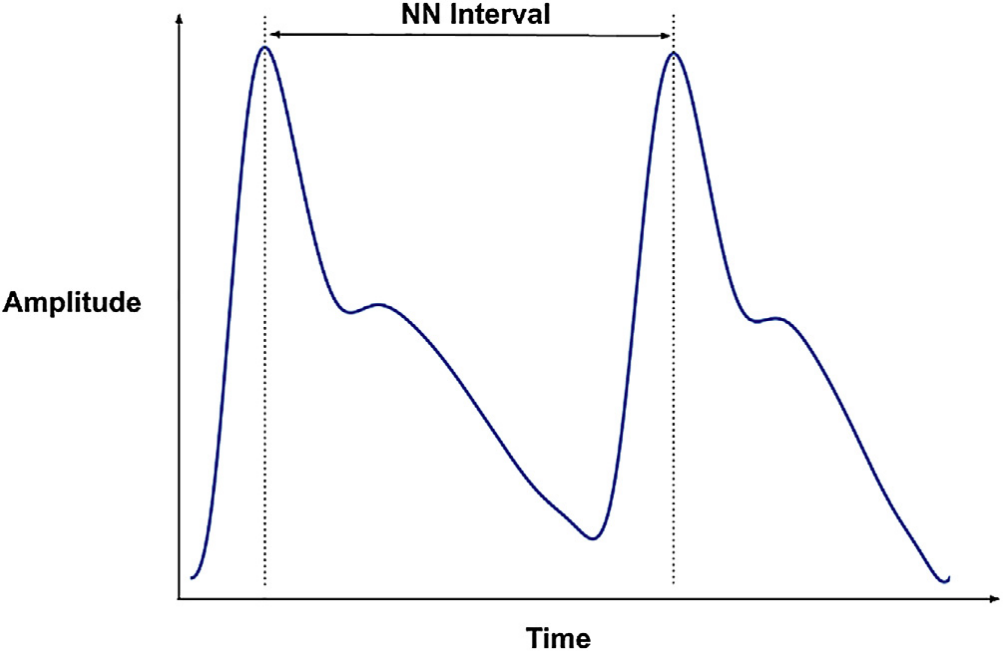
PPG signal breakdown.

In a typical PPG signal, the Normal-to-Normal interval (NN) pertains to the duration between successive peaks (Fig. 1). The heart rate variability (HRV) analyses the time differences between these peaks in different ways and domains presented in the next table. The list of the features and their description has been taken from Ma et al. research [5].

**Table 16:** Samsung/hrv_1min.csv and samsung/hrv_5min.csv.

Column name	Type of HRV Feature	Description	Range	Type of variable
timestamp	-	Starting time of the data segment	-	Timestamp
hrv_meannn	Time Domain	Mean of normal-to-normal interval (NN)	-	Number (milliseconds)
hrv_sdnn	Time Domain	The standard deviation (SD) of the NN	-	Number (milliseconds)
hrv_rmssd	Time Domain	Root mean square of successive NN interval differences	-	Number (milliseconds)
hrv_sdsd	Time Domain	SD of the successive differences between NN	-	Number (milliseconds)
hrv_cvnn	Time Domain	The standard deviation of the NN intervals (hrv_sdnn) divided by the mean of the NN intervals (hrv_meannn)	-	Number (%)
hrv_cvsd	Time Domain	hrv_sdnn divided by hrv_meannn	-	Number (%)
hrv_mediannn	Time Domain	Median of NN	-	Number (milliseconds)
hrv_madnn	Time Domain	Median absolute deviation of NN	-	Number (milliseconds)
hrv_mcvnn	Time Domain	Median absolute deviation of NN (hrv_madnn) divided by Median of NN (hrv_mediannn)	-	Number (milliseconds)
hrv_iqrnn	Time Domain	Interquartile range of NN	-	Number (milliseconds)
hrv_prc80nn	Time Domain	The 80th percentile of the NN intervals	-	Number (milliseconds)
hrv_pnn50	Time Domain	Percentage of successive NN that differ by more than 50ms	-	Number (%)
hrv_pnn20	Time Domain	Percentage of successive NN that differ by more than 20ms	-	Number (%)
hrv_minnn	Time Domain	The minimum of the NN intervals	-	Number (milliseconds)
hrv_maxnn	Time Domain	The maximum of the NN intervals	-	Number (milliseconds)
hrv_tinn	Time Domain	Baseline width of the NN distribution obtained by triangular interpolation	-	Number
hrv_hti	Time Domain	Total number of NN divided by the height of the NN histogram	-	Number
hrv_ulf	Time Domain	The spectral power of ultra low frequencies (.0033 Hz).	-	Number (ms^2^)
hrv_vlf	Frequency Domain	The spectral power of very low frequencies (.0033 to .04 Hz)	-	Number (ms^2^)
hrv_lf	Frequency Domain	The spectral power of low frequencies (by default, .04 to .15 Hz)	-	Number (ms^2^)
hrv_hf	Frequency Domain	The spectral power of high frequencies (by default, .15 to .4 Hz)	-	Number (ms^2^)
hrv_vhf	Frequency Domain	Absolute power of the very-high-frequency band (0.4–0.5Hz)	-	Number (ms^2^)
hrv_lfhf	Frequency Domain	The ratio obtained by dividing the low frequency power by the high frequency power	-	Number
hrv_lfn	Frequency Domain	The normalized low frequency, low-frequency power divided by the total power	-	Number
hrv_hfn	Frequency Domain	The normalized high frequency, high-frequency power divided by the total power	-	Number
hrv_lnhf	Frequency Domain	The log transformed hrv_hf	-	Number (ms^2^)
hrv_sd1	Non-linear Metric	Index of short-term NN fluctuation, the semi-short axis of the fitted ellipse in the Poincaré plot	-	Number
hrv_sd2	Non-linear Metric	Index of long-term NN fluctuation, the semi-long axis of the fitted ellipse in the Poincaré plot	-	Number
hrv_sd1sd2	Non-linear Metric	Ratio of SD1-to-SD2	-	Number
hrv_s	Non-linear Metric	Area of the fitted ellipse in the Poincaré plot	-	Number
hr	-	Heart rate	-	Number (beats/minute)

hrv_1min.csv and hrv_5min.csv.

#### 2.3.4 Assessment

This directory contains two comma separated vector files “events.csv” and “surveys.csv” respectively presented in Tables 17 and 18. The surveys.csv contains the accumulation of all the surveys that were asked from the participant through different stages of the study. in the events.csv we asked the participants what they thought about different events that have happened during their participation in the study and how these events affected them. These surveys were collected in person before the COVID-19 lockdown and then online through Qualtrics software. events.csv.

**Table 17:** Assessment/events.csv.

Column name	Description	Range	Unit
start_timestamp	The timestamp that the participant has started filling out the survey.	-	Timestamp (milliseconds)
end_timestamp	The timestamp that the participant has finished filling out the survey.	-	Timestamp (milliseconds)
stage	Which stage this survey was taken for. • T1 (Enrollment) • T2 • T3 • T4 • T5 • Final (Exit)	On of: • T1 to T5 • Final	Text
progress	How far the participant answered the survey questionnaires.	0-100	Number
duration_sec	The time it took the participant to finish the survey.	-	Number (seconds)
finished	Did the participant finish the survey	True/False	Boolean
rel_length	Answer to “How long have you and your partner been romantically involved?” Choices: • Less than 1 month • Between 1-3 months • Between 3-6 months • Between 6-12 months • More than 12 months	On of: • Less than 1 month • Between 1-3 months • Between 3-6 months • Between 6-12 months • More than 12 months	Text
aim_1	Answer to “Personicle meets my approval.” Choices: • Completely Disagree • Disagree • Neither Agree Nor Disagree • Agree • Completely Agree	One of: • Completely Disagree • Disagree • Neither Agree Nor Disagree • Agree • Completely Agree	Text
aim_2	Answer to “Personicle is appealing to me.” Choices: • Completely Disagree • Disagree • Neither Agree Nor Disagree • Agree • Completely Agree	One of: • Completely Disagree • Disagree • Neither Agree Nor Disagree • Agree • Completely Agree	Text
aim_3	Answer to “I like Personicle.” Choices: • Completely Disagree • Disagree • Neither Agree Nor Disagree • Agree • Completely Agree	One of: • Completely Disagree • Disagree • Neither Agree Nor Disagree • Agree • Completely Agree	Text
aim_4	Answer to “I welcome Personicle.” Choices: • Completely Disagree • Disagree • Neither Agree Nor Disagree • Agree • Completely Agree	One of: • Completely Disagree • Disagree • Neither Agree Nor Disagree • Agree • Completely Agree	Text
bc_1	Answer to “In the past week, how much time have you spent seeking out information about the coronavirus each day?” • No time • Less than 30 minutes • 30 minutes-1 hour • 1-4 hours • 4-8 hours • 8+ hours	One of • No time • Less than 30 minutes • 30 minutes-1 hour • 1-4 hours • 4-8 hours • 8+ hours	Text
bc_2	Answer to “Which of these sources do you get your news from most frequently?” Choices: • In person communication doctor/health care provider • in person communication - friends/family • Television news • Online - social media (e.g., Facebook, Twitter) • Online - government/healthcare websites (e.g., CDC.gov) • Online - news outlets • Other	One of • In person communication doctor/health care provider • in person communication - friends/family • Television news • Online - social media (e.g., Facebook, Twitter) • Online - government/healthcare websites (e.g., CDC.gov) • Online - news outlets • Other	Text
bc_3_1	Answer to “In the past week, how much have you made it a priority to get enough sleep?” Choices: • Not at all • A little bit • Some • A lot • As much as possible	One of • Not at all • A little bit • Some • A lot • As much as possible	Text
bc_3_2	Answer to “In the past week, how much have you made it a priority to eat nutritiously?” Choices • Not at all • A little bit • Some • A lot • As much as possible	One of • Not at all • A little bit • Some • A lot • As much as possible	Text
bc_3_3	Answer to “In the past week, how much have you made it a priority to exercise regularly?” Choices • Not at all • A little bit • Some • A lot • As much as possible	One of • Not at all • A little bit • Some • A lot • As much as possible	Text
bc_3_4	Answer to “In the past week, how much have you made it a priority to avoid smoking?” Choices • Not at all • A little bit • Some • A lot • As much as possible	One of • Not at all • A little bit • Some • A lot • As much as possible	Text
bc_3_5	Answer to “In the past week, how much have you made it a priority to take extra vitamins or supplements?” Choices • Not at all • A little bit • Some • A lot • As much as possible	One of • Not at all • A little bit • Some • A lot • As much as possible	Text
bc_3_6	Answer to “In the past week, how much have you made it a priority to wash your hands?” Choices • Not at all • A little bit • Some • A lot • As much as possible	One of • Not at all • A little bit • Some • A lot • As much as possible	Text
bc_4_1	Answer to “In the past week, how much have you made it a priority to wash your hands?” Choices • Not at all • A little bit • Some • A lot • As much as possible	One of • Not at all • A little bit • Some • A lot • As much as possible	Text
bc_4_2	Answer to “In the past week, how much have you made it a priority to wash your hands for at least 20 seconds?” Choices • Not at all • A little bit • Some • A lot • As much as possible	One of • Not at all • A little bit • Some • A lot • As much as possible	Text
bc_4_3	Answer to “In the past week, how much have you made it a priority to stay home?” Choices • Not at all • A little bit • Some • A lot • As much as possible	One of • Not at all • A little bit • Some • A lot • As much as possible	Text
bc_4_4	Answer to “In the past week, how much have you made it a priority to clean and disinfect your home?” Choices • Not at all • A little bit • Some • A lot • As much as possible	One of • Not at all • A little bit • Some • A lot • As much as possible	Text
bc_4_5	Answer to “In the past week, how much have you made it a priority to use antibacterial products?” Choices • Not at all • A little bit • Some • A lot • As much as possible	One of • Not at all • A little bit • Some • A lot • As much as possible	Text
bc_4_6	Answer to “In the past week, how much have you made it a priority to get fresh air/increase ventilation?” Choices • Not at all • A little bit • Some • A lot • As much as possible	One of • Not at all • A little bit • Some • A lot • As much as possible	Text
bc_4_7	Answer to “In the past week, how much have you made it a priority to not touch your face?” Choices • Not at all • A little bit • Some • A lot • As much as possible	One of • Not at all • A little bit • Some • A lot • As much as possible	Text
bc_4_8	Answer to “In the past week, how much have you made it a priority to avoid shaking hands with people?” Choices • Not at all • A little bit • Some • A lot • As much as possible	One of • Not at all • A little bit • Some • A lot • As much as possible	Text
bc_4_9	Answer to “In the past week, how much have you made it a priority to avoid any physical contact with people?” Choices • Not at all • A little bit • Some • A lot • As much as possible	One of • Not at all • A little bit • Some • A lot • As much as possible	Text
bc_4_10	Answer to “In the past week, how much have you made it a priority to wear a mask of any kind?” Choices • Not at all • A little bit • Some • A lot • As much as possible	One of • Not at all • A little bit • Some • A lot • As much as possible	Text
bc_4_11	Answer to “In the past week, how much have you made it a priority to wear an N-95 or higher (health grade) mask?” Choices • Not at all • A little bit • Some • A lot • As much as possible	One of • Not at all • A little bit • Some • A lot • As much as possible	Text
bc_4_12	Answer to “In the past week, how much have you made it a priority toavoid asian food/restaurants?” Choices • Not at all • A little bit • Some • A lot • As much as possible	One of • Not at all • A little bit • Some • A lot • As much as possible	Text
bc_4_13	Answer to “In the past week, how much have you made it a priority toavoid food prepared by someone you do not know?” Choices • Not at all • A little bit • Some • A lot • As much as possible	One of • Not at all • A little bit • Some • A lot • As much as possible	Text
bc_4_14	Answer to “In the past week, how much have you made it a priority toprepare for being quarantined?” Choices • Not at all • A little bit • Some • A lot • As much as possible	One of • Not at all • A little bit • Some • A lot • As much as possible	Text
bc_5	Answer to “For the above actions what was your primary motivation?” Choices • Only to protect myself • Mostly to protect myself • Mostly to protect others • Only to protect others	One of • Only to protect myself • Mostly to protect myself • Mostly to protect others • Only to protect others	Text
bc_6	Answer to “If there were a vaccine for the coronavirus, how likely would you be to get vaccinated?” Choices • Not at all likely • A little bit likely • Somewhat likely • Very likely • Extremely likely	One of • Not at all likely • A little bit likely • Somewhat likely • Very likely • Exremely likely	Text
bc_7	Answer to “Even if you are at low risk, would you receive the coronavirus vaccine to protect others around you?” Choices • Not at all likely • A little bit likely • Somewhat likely • Very likely • Extremely likely	One of • Not at all likely • A little bit likely • Somewhat likely • Very likely • Extremely likely	Text
bc_8	Answer to “In the past week, have you voluntarily self-quarantined yourself for any period of time” Choices • Yes • No	One of • Yes • No	Text
bc_9	Answer to “In the past week, have you contacted a health care provider (i.e., doctor or nurse) for any reason?” Choices • Yes • No	One of • Yes • No	Text
bc_10	Answer to “Have you contacted a health provider (i.e., doctor or nurse) with concerns about the coronavirus?” Choices • Yes • No	One of • Yes • No	Text
bc_11	Answer to “Have you been diagnosed with the coronavirus?” Choices Yes No	One of Yes No	Text
bc_12	Answer to “Have you been hospitalized for coronavirus?” Choices • Yes • No	One of • Yes • No	Text
bc_13	Answer to “Have you been quarantined for coronavirus?” Choices • Yes • No	One of • Yes • No	Text
bc_14	Prompt to question “If you have been hospitalized for coronavirus?=yes” Answer to “When were you hospitalized.” Format: (MM/DD/YYYY) **This field is removed to protect participants’ privacy.**	-	Text
bc_15	Prompt to question “If you have been quarantined for coronavirus?=yes” Answer to “When were you quarantined.” Format: (MM/DD/YYYY) **This field is removed to protect participants’ privacy.**	-	Text
bc_16	Answer to “For how long were you quarantined?” Choices • Less than 1 day • 1 day to 1 week • 1-2 weeks • Longer than 2 weeks	One of • Less than 1 day • 1 day to 1 week • 1-2 weeks • Longer than 2 weeks	Text
bc_17	Answer to “How much has your work/professional life been affected by the outbreak of the coronavirus?” Choices • None • A little • A moderate amount • A lot • A great deal • Not applicable	One of • None • A little • A moderate amount • A lot • A great deal • Not applicable	Text
bc_18	Answer to “How much has your personal/family life been affected by the outbreak of the coronavirus?” Choices • Not at all • A little • A moderate amount • A lot • A great deal	One of • Not at all • A little • A moderate amount • A lot • A great deal	Text
bc_19	Answer to “How much have you been financially affected by the coronavirus outbreak?” Choices • Not at all • A little • A moderate amount • A lot • A great deal	One of • Not at all • A little • A moderate amount • A lot • A great deal	Text
bct **(not a real column. this column is only providing information for bct_1 to bct_3)**	Prompt to questions “Please indicate how much you agree with the statement by selecting the appropriate response below” Choices • 1 (Stronly disagree) • 2 • 3 • 4 • 5 (Strongly agree)	N/A	N/A
bct_1	Answer to “A foreign government deliberately spread the coronavirus as a bioweapon.” Choices • 1 (Strongly disagree) • 2 • 3 • 4 • 5 (Strongly agree)	One of • 1 (Strongly disagree) • 2 • 3 • 4 • 5 (Strongly agree)	Number
bct_2	Answer to “Experts and media outlets are exaggerating the threat of coronavirus to weaponize it for political purposes.” Choices • 1 (Strongly disagree) • 2 • 3 • 4 • 5 (Strongly agree)	One of • 1 (Strongly disagree) • 2 • 3 • 4 • 5 (Strongly agree)	Number
bct_3	Answer to “Vaccines are harmful, and this fact is covered up.” Choices • 1 (Strongly disagree) • 2 • 3 • 4 • 5 (Strongly agree)	One of • 1 (Strongly disagree) • 2 • 3 • 4 • 5 (Strongly agree)	Number
bdi **(not a real column. this column is only providing information for bdi_1 to bdi_21)**	Prompt to question “Please read each group of statements carefully, and then pick out the one statement in each group that best describes the way you have been feeling during the past two weeks, including today.”	N/A	N/A
bdi_1	Answer to “Sadness” Choices: • I do not feel sad • I feel sad much of the time. • I am sad all the time. • I am so sad or unhappy that I can't stand it	One of: • I do not feel sad • I feel sad much of the time. • I am sad all the time. • I am so sad or unhappy that I can't stand it	Text
bdi_2	Answer to “Pessimism” Choices • I am not discouraged about my future • I feel sad much of the time • I am sad all the time • I am so sad or unhappy that I can't stand it	One of • I am not discouraged about my future • I feel sad much of the time • I am sad all the time • I am so sad or unhappy that I can't stand it	Text
bdi_3	Answer to “Past Failure” Choices • I do not feel like a failure • I have failed more than I should have • As I look back, I see a lot of failures • I feel I am a total failure as a person	One of • I do not feel like a failure • I have failed more than I should have • As I look back, I see a lot of failures • I feel I am a total failure as a person	Text
bdi_4	Answer to “Loss of Pleasure” Choices • I get as much pleasure as I ever did from the things I enjoy • I don't enjoy things as much as I used to • I get very little pleasure form the things I used to enjoy	One of • I get as much pleasure as I ever did from the things I enjoy • I don't enjoy things as much as I used to • I get very little pleasure form the things I used to enjoy	Text
bdi_5	Answer to “Guilty Feelings” Choices • I don't feel particularly guilty • I feel guilty over many things I have done or should have done • I feel quite guilty most of the time • I feel guilty all the time	One of • I don't feel particularly guilty • I feel guilty over many things I have done or should have done • I feel quite guilty most of the time • I feel guilty all the time	Text
bdi_6	Answer to “Punishment Feelings” Choices • I don't feel I am being punished • I feel I may be punished • I expect to be punished • I feel I am being punished	One of • I don't feel I am being punished • I feel I may be punished • I expect to be punished • I feel I am being punished	Text
bdi_7	Answer to “Self-Dislike” Choices • I feel the same about myself as ever • I have lost confidence i myself • I am disappointed in myself • I dislike myself	One of • I feel the same about myself as ever • I have lost confidence i myself • I am disappointed in myself • I dislike myself	Text
bdi_8	Answer to “Self-Criticalness” Choices • I don't criticize or blame myself more than usual • I am more critical of myself than I used to be • I criticize myself for all of my faults • I blame myself for everything bad that happens	One of • I don't criticize or blame myself more than usual • I am more critical of myself than I used to be • I criticize myself for all of my faults • I blame myself for everything bad that happens	Text
bdi_9	Answer to “Suicidal Thoughts or Wishes” Choices • I don't have any thoughts of killing myself • I have thoughts of killing myself, but I would not carry them out • I would like to kill myself • I would kill myself if I had the chance **This field is only available upon request to protect participants’ privacy.**	One of • I don't have any thoughts of killing myself • I have thoughts of killing myself, but I would not carry them out • I would like to kill myself • I would kill myself if I had the chance	Text
bdi_10	Answer to “Crying” Choices • I don't cry anymore than I used to • I cry more than I used to • I cry over every little thing • I feel like crying, but I can't	One of • I don't cry anymore than I used to • I cry more than I used to • I cry over every little thing • I feel like crying, but I can't	Text
bdi_11	Answer to “Agitation” Choices • I am no more restless or wound up than usual • I feel more restless or wound up than usual • I am so restless or agitated that it's hard to stay still • I am so restless or agitated that I have to keep moving or doing something	One of • I am no more restless or wound up than usual • I feel more restless or wound up than usual • I am so restless or agitated that it's hard to stay still • I am so restless or agitated that I have to keep moving or doing something	Text
bdi_12	Answer to “Loss of Interest” Choices • I have not lost interest in other people or activities • I am less interested in other people or things than before • I have lost most of my interest in other people or things • It's hard to get interested in anything	One of • I have not lost interest in other people or activities • I am less interested in other people or things than before • I have lost most of my interest in other people or things • It's hard to get interested in anything	Text
bdi_13	Answer to “Indecisiveness” Choices • I make decisions about as well as ever • I find it more difficult to make decisions than usual • I have much greater difficulty in making decisions than I used to • I have trouble making any decisions	One of • I make decisions about as well as ever • I find it more difficult to make decisions than usual • I have much greater difficulty in making decisions than I used to • I have trouble making any decisions	Text
bdi_14	Answer to “Worthlessness” Choices • I do not feel I am worthless • I don't consider myself as worthwhile and useful as I used to • I feel more worthless as compared to other people • I feel utterly worthless	One of • I do not feel I am worthless • I don't consider myself as worthwhile and useful as I used to • I feel more worthless as compared to other people • I feel utterly worthless	Text
bdi_15	Answer to “Loss of Energy” Choices • I have as much energy as ever • I have less energy than I used to have • I don't have enough energy to do very much • I don't have enough energy to do anything	One of • I have as much energy as ever • I have less energy than I used to have • I don't have enough energy to do very much • I don't have enough energy to do anything	Text
bdi_16	Answer to “Changes in Sleeping Pattern” Choices • I have not experienced any change in my sleeping pattern • I sleep somewhat more than usual • I sleep somewhat less than usual • I sleep a lot more than usual • I sleep a lot less than usual • I sleep most of the day • I wake up 1-2 hours early and can't get back to sleep	One of • I have not experienced any change in my sleeping pattern • I sleep somewhat more than usual • I sleep somewhat less than usual • I sleep a lot more than usual I sleep a lot less than usual • I sleep most of the day • I wake up 1-2 hours early and can't get back to sleep	
bdi_17	Answer to “Irritability” Choices • I am no more irritable than usual • I am more irritable than usual • I am much more irritable than usual • I am irritable all the time	One of • I am no more irritable than usual • I am more irritable than usual • I am much more irritable than usual • I am irritable all the time	Text
bdi_18	Answer to “Changes in Appetite” Choices • I have not experienced any change in my appetite • My appetite is somewhat less than usual • My appetite is somewhat greater than usual • My appetite is much less than before • My appetite is much greater than usual • I have no appetite at all • I crave food all the time	One of • I have not experienced any change in my appetite • My appetite is somewhat less than usual • My appetite is somewhat greater than usual • My appetite is much less than before • My appetite is much greater than usual • I have no appetite at all • I crave food all the time	
bdi_19	Answer to “Concetration Difficulty” Choices • I can concentrate as well as ever • I can't concentrate as well as usual • It's hard to keep my mind on anything for very long • I find I can't concentrate on anything	One of • I can concentrate as well as ever • I can't concentrate as well as usual • It's hard to keep my mind on anything for very long • I find I can't concentrate on anything	Text
bdi_20	Answer to “Tiredness or Fatigue” Choices • I am no more tired or fatigued than usual • I get more tired or fatigued more easily than usual • I am too tired or fatigued to do a lot of things I used to do • I am too tired or fatigued to do most of the things	One of • I am no more tired or fatigued than usual • I get more tired or fatigued more easily than usual • I am too tired or fatigued to do a lot of things I used to do • I am too tired or fatigued to do most of the things	Text
bdi_21	Answer to “Loss of Interest in Sex” Choices • I have not noticed any recent change in my interest in sex • I am less interested in sex than I used to be • I am much less interested in sex now • I have lost interest in sex completely	One of • I have not noticed any recent change in my interest in sex • I am less interested in sex than I used to be • I am much less interested in sex now • I have lost interest in sex completely	Text
bsi **(not a real column. This column is only providing information for bsi_1 to bsi_53)**	Prompt to question “During the last 7 days, how much were you distressed by..” Choices • Not at all • A little bit • Moderately • Quite a bit • Extremely	N/A	N/A
bsi_1	Answer to “Nervousness or shakiness inside.” Choices • Not at all • A little bit • Moderately • Quite a bit • Extremely	One of • Not at all • A little bit • Moderately • Quite a bit • Extremely	Text
bsi_2	Answer to “Faintness or dizziness.” Choices • Not at all • A little bit • Moderately • Quite a bit • Extremely	One of • Not at all • A little bit • Moderately • Quite a bit • Extremely	Text
bsi_3	Answer to “The idea that someone else can control your thoughts.” Choices • Not at all • A little bit • Moderately • Quite a bit • Extremely	One of • Not at all • A little bit • Moderately • Quite a bit • Extremely	Text
bsi_4	Answer to “Feeling others are to blame for most of your troubles.” Choices • Not at all • A little bit • Moderately • Quite a bit • Extremely	One of • Not at all • A little bit • Moderately • Quite a bit • Extremely	Text
bsi_5	Answer to “Trouble remembering things.” Choices • Not at all • A little bit • Moderately • Quite a bit • Extremely	One of • Not at all • A little bit • Moderately • Quite a bit • Extremely	Text
bsi_6	Answer to “Feeling easily annoyed or irritated.” Choices • Not at all • A little bit • Moderately • Quite a bit • Extremely	One of • Not at all • A little bit • Moderately • Quite a bit • Extremely	Text
bsi_7	Answer to “Pains in the heart or chest.” Choices • Not at all • A little bit • Moderately • Quite a bit • Extremely	One of • Not at all • A little bit • Moderately • Quite a bit • Extremely	Text
bsi_8	Answer to “Feeling afraid in open space.” Choices • Not at all • A little bit • Moderately • Quite a bit • Extremely	One of • Not at all • A little bit • Moderately • Quite a bit • Extremely	Text
bsi_9	Answer to “Thoughts of ending your life.” Choices • Not at all • A little bit • Moderately • Quite a bit • Extremely	One of • Not at all • A little bit • Moderately • Quite a bit • Extremely	Text
bsi_10	Answer to “Feeling that most people cannot be trusted.” Choices • Not at all • A little bit • Moderately • Quite a bit • Extremely	One of • Not at all • A little bit • Moderately • Quite a bit • Extremely	Text
bsi_11	Answer to “Poor appetite.” Choices • Not at all • A little bit • Moderately • Quite a bit • Extremely	One of • Not at all • A little bit • Moderately • Quite a bit • Extremely	Text
bsi_12	Answer to “Suddenly scared for no reason.” Choices • Not at all • A little bit • Moderately • Quite a bit • Extremely	One of • Not at all • A little bit • Moderately • Quite a bit • Extremely	Text
bsi_13	Answer to “Temper outbursts that you could not control.” Choices • Not at all • A little bit • Moderately • Quite a bit • Extremely	One of • Not at all • A little bit • Moderately • Quite a bit • Extremely	Text
bsi_14	Answer to “Feeling lonely even when you are with people.” Choices • Not at all • A little bit • Moderately • Quite a bit • Extremely	One of • Not at all • A little bit • Moderately • Quite a bit • Extremely	Text
bsi_15	Answer to “Feeling blocked in getting things done.” Choices • Not at all • A little bit • Moderately • Quite a bit • Extremely	One of • Not at all • A little bit • Moderately • Quite a bit • Extremely	Text
bsi_16	Answer to “Feeling lonely.” Choices • Not at all • A little bit • Moderately • Quite a bit • Extremely	One of • Not at all • A little bit • Moderately • Quite a bit • Extremely	Text
bsi_17	Answer to “Feeling blue.” Choices • Not at all • A little bit • Moderately • Quite a bit • Extremely	One of • Not at all • A little bit • Moderately • Quite a bit • Extremely	Text
bsi_18	Answer to “Feeling no interest in things.” Choices • Not at all • A little bit • Moderately • Quite a bit • Extremely	One of • Not at all • A little bit • Moderately • Quite a bit • Extremely	Text
bsi_19	Answer to “Feeling fearful.” Choices • Not at all • A little bit • Moderately • Quite a bit • Extremely	One of • Not at all • A little bit • Moderately • Quite a bit • Extremely	Text
bsi_20	Answer to “Your feelings being easily hurt.” Choices • Not at all • A little bit • Moderately • Quite a bit • Extremely	One of • Not at all • A little bit • Moderately • Quite a bit • Extremely	Text
bsi_21	Answer to “Feeling that people are unfriendly or dislike you.” Choices • Not at all • A little bit • Moderately • Quite a bit • Extremely	One of • Not at all • A little bit • Moderately • Quite a bit • Extremely	Text
bsi_22	Answer to “Feeling inferior to others.” Choices • Not at all • A little bit • Moderately • Quite a bit • Extremely	One of • Not at all • A little bit • Moderately • Quite a bit • Extremely	Text
bsi_23	Answer to “Nausea or upset stomach.” Choices • Not at all • A little bit • Moderately • Quite a bit • Extremely	One of • Not at all • A little bit • Moderately • Quite a bit • Extremely	Text
bsi_24	Answer to “Feeling that you are watched or talked about by others.” Choices • Not at all • A little bit • Moderately • Quite a bit • Extremely	One of • Not at all • A little bit • Moderately • Quite a bit • Extremely	Text
bsi_25	Answer to “Trouble falling asleep.” Choices • Not at all • A little bit • Moderately • Quite a bit • Extremely	One of • Not at all • A little bit • Moderately • Quite a bit • Extremely	Text
bsi_26	Answer to “Having to check and what you do.” Choices • Not at all • A little bit • Moderately • Quite a bit • Extremely	One of • Not at all • A little bit • Moderately • Quite a bit • Extremely	Text
bsi_27	Answer to “Difficulty making decisions.” Choices • Not at all • A little bit • Moderately • Quite a bit • Extremely	One of • Not at all • A little bit • Moderately • Quite a bit • Extremely	Text
bsi_28	Answer to “Feeling afraid to travel on buses, subways, or trains.” Choices • Not at all • A little bit • Moderately • Quite a bit • Extremely	One of • Not at all • A little bit • Moderately • Quite a bit • Extremely	Text
bsi_29	Answer to “Trouble getting your breath.” Choices • Not at all • A little bit • Moderately • Quite a bit • Extremely	One of • Not at all • A little bit • Moderately • Quite a bit • Extremely	Text
bsi_30	Answer to “Hot or cold spells.” Choices • Not at all • A little bit • Moderately • Quite a bit • Extremely	One of • Not at all • A little bit • Moderately • Quite a bit • Extremely	Text
bsi_31	Answer to “Having to avoid certain things, places, or activities because they frighten you.” Choices • Not at all • A little bit • Moderately • Quite a bit • Extremely	One of • Not at all • A little bit • Moderately • Quite a bit • Extremely	Text
bsi_32	Answer to “Your mind going blank.” Choices • Not at all • A little bit • Moderately • Quite a bit • Extremely	One of • Not at all • A little bit • Moderately • Quite a bit • Extremely	Text
bsi_33	Answer to “Numbness or tingling in parts of your body.” Choices • Not at all • A little bit • Moderately • Quite a bit • Extremely	One of • Not at all • A little bit • Moderately • Quite a bit • Extremely	Text
bsi_34	Answer to “The idea that you should be punished for your sins.” Choices • Not at all • A little bit • Moderately • Quite a bit • Extremely **This field is only available upon request to protect participants’ privacy.**	One of • Not at all • A little bit • Moderately • Quite a bit • Extremely	Text
bsi_35	Answer to “Feeling hopeless about the future.” Choices • Not at all • A little bit • Moderately • Quite a bit • Extremely	One of • Not at all • A little bit • Moderately • Quite a bit • Extremely	Text
bsi_36	Answer to “Trouble concentrating.” Choices • Not at all • A little bit • Moderately • Quite a bit • Extremely	One of • Not at all • A little bit • Moderately • Quite a bit • Extremely	Text
bsi_37	Answer to “Feeling weak in parts of your body.” Choices • Not at all • A little bit • Moderately • Quite a bit • Extremely	One of • Not at all • A little bit • Moderately • Quite a bit • Extremely	Text
bsi_38	Answer to “Feeling tense or keyed up.” Choices • Not at all • A little bit • Moderately • Quite a bit • Extremely	One of • Not at all • A little bit • Moderately • Quite a bit • Extremely	Text
bsi_39	Answer to “Thoughts of death or dying.” Choices • Not at all • A little bit • Moderately • Quite a bit • Extremely **This field is only available upon request to protect participants’ privacy.**	One of • Not at all • A little bit • Moderately • Quite a bit • Extremely	Text
bsi_40	Answer to “Having urges to beat, injure, or harm someone.” Choices • Not at all • A little bit • Moderately • Quite a bit • Extremely	One of • Not at all • A little bit • Moderately • Quite a bit • Extremely	Text
bsi_41	Answer to “Having urges to break or smash things.” Choices • Not at all • A little bit • Moderately • Quite a bit • Extremely	One of • Not at all • A little bit • Moderately • Quite a bit • Extremely	Text
bsi_42	Answer to “Feeling very self-conscious with others.” Choices • Not at all • A little bit • Moderately • Quite a bit • Extremely	One of • Not at all • A little bit • Moderately • Quite a bit • Extremely	Text
bsi_43	Answer to “Feeling uneasy in crowds.” Choices • Not at all • A little bit • Moderately • Quite a bit • Extremely	One of • Not at all • A little bit • Moderately • Quite a bit • Extremely	Text
bsi_44	Answer to “Never feeling close to another person.” Choices • Not at all • A little bit • Moderately • Quite a bit • Extremely	One of • Not at all • A little bit • Moderately • Quite a bit • Extremely	Text
bsi_45	Answer to “Spells of terror or panic.” Choices • Not at all • A little bit • Moderately • Quite a bit • Extremely	One of • Not at all • A little bit • Moderately • Quite a bit • Extremely	Text
bsi_46	Answer to “Getting into frequent arguments.” Choices • Not at all • A little bit • Moderately • Quite a bit • Extremely	One of • Not at all • A little bit • Moderately • Quite a bit • Extremely	Text
bsi_47	Answer to “Feeling nervous when you are left alone.” Choices • Not at all • A little bit • Moderately • Quite a bit • Extremely	One of • Not at all • A little bit • Moderately • Quite a bit • Extremely	Text
bsi_48	Answer to “Others not giving you proper credit for your achievements.” Choices • Not at all • A little bit • Moderately • Quite a bit • Extremely	One of • Not at all • A little bit • Moderately • Quite a bit • Extremely	Text
bsi_49	Answer to “Feeling so restless you couldn't sit still.” Choices • Not at all • A little bit • Moderately • Quite a bit • Extremely	One of • Not at all • A little bit • Moderately • Quite a bit • Extremely	Text
bsi_50	Answer to “Feelings of worthlessness.” Choices • Not at all • A little bit • Moderately • Quite a bit • Extremely	One of • Not at all • A little bit • Moderately • Quite a bit • Extremely	Text
bsi_51	Answer to “Feeling that people will take advantage of you if you let them.” Choices • Not at all • A little bit • Moderately • Quite a bit • Extremely	One of • Not at all • A little bit • Moderately • Quite a bit • Extremely	Text
bsi_52	Answer to “Feelings of guilt.” Choices • Not at all • A little bit • Moderately • Quite a bit • Extremely	One of • Not at all • A little bit • Moderately • Quite a bit • Extremely	Text
bsi_53	Answer to “The idea that something is wrong with your mind.” Choices • Not at all • A little bit • Moderately • Quite a bit • Extremely	One of • Not at all • A little bit • Moderately • Quite a bit • Extremely	Text
cms **(not a real column. This column is only providing information for cms_1 to cms_7)**	Prompt to question “Please indicate how much you agree or disagree with each statement. (If currently single you may relate the following answers to a past or ideal relationship)” Choices • Strongly Disagree • Disagree • Agree • Strongly Agree	N/A	N/A
cms_1	Answer to “I believe that in the couple relationship not only my needs are important” Choices • Strongly Disagree • Disagree • Agree • Strongly Agree	One of • Strongly Disagree • Disagree • Agree • Strongly Agree	Text
cms_2	Answer to “Usually, I leave a discussion without giving any reason” Choices • Strongly Disagree • Disagree • Agree • Strongly Agree	One of • Strongly Disagree • Disagree • Agree • Strongly Agree	Text
cms_3	Answer to “I think it is better to solve a conflicting situation instead of ignoring it” Choices • Strongly Disagree • Disagree • Agree • Strongly Agree	One of • Strongly Disagree • Disagree • Agree • Strongly Agree	Text
cms_4	Answer to “I try to collaborate with my partner actively in view of solving a conflicting situation” Choices • Strongly Disagree • Disagree • Agree • Strongly Agree	One of • Strongly Disagree • Disagree • Agree • Strongly Agree	Text
cms_5	Answer to “I believe it is necessary to face the discussion with my partner without running away” Choices • Strongly Disagree • Disagree • Agree • Strongly Agree	One of • Strongly Disagree • Disagree • Agree • Strongly Agree	Text
cms_6	Answer to “I am more careful with satisfying my wishes than those of my partner” Choices • Strongly Disagree • Disagree • Agree • Strongly Agree	One of • Strongly Disagree • Disagree • Agree • Strongly Agree	Text
cms_7	Answer to “I believe that in a conflicting situation both partners should move a step toward the other” Choices • Strongly Disagree • Disagree • Agree • Strongly Agree	One of • Strongly Disagree • Disagree • Agree • Strongly Agree	Text
cms_partner **(not a real column. This column is only providing information for cms_partner_1 to cms_partner_7)**	Prompt to question “Please indicate how much you agree or disagree with each statement. (If currently single you may relate the following answers to a past or ideal relationship)” Choices • Strongly Disagree • Disagree • Agree • Strongly Agree	N/A	N/A
cms_partner_1	Answer to “My partner believes tha tin the couple relationship not only their needs are important” Choices • Strongly Disagree • Disagree • Agree • Strongly Agree	One of • Strongly Disagree • Disagree • Agree • Strongly Agree	Text
cms_partner_2	Answer to “Usually, my partner leaves a discussion without giving any reason” Choices • Strongly Disagree • Disagree • Agree • Strongly Agree	One of • Strongly Disagree • Disagree • Agree • Strongly Agree	Text
cms_partner_3	Answer to “My partner thinks it is better to solve a conflicting situation instead of ignoring it” Choices • Strongly Disagree • Disagree • Agree • Strongly Agree	One of • Strongly Disagree • Disagree • Agree • Strongly Agree	Text
cms_partner_4	Answer to “My partner tries to collaborate with me actively in view of solving a conflicting situation” Choices • Strongly Disagree • Disagree • Agree • Strongly Agree	One of • Strongly Disagree • Disagree • Agree • Strongly Agree	Text
cms_partner_5	Answer to “My partner believes it is necessary to fae the discussion with me without running away” Choices • Strongly Disagree • Disagree • Agree • Strongly Agree	One of • Strongly Disagree • Disagree • Agree • Strongly Agree	Text
cms_partner_6	Answer to “My partner is more careful with satisfying his/her wishes than mine” Choices • Strongly Disagree • Disagree • Agree • Strongly Agree	One of • Strongly Disagree • Disagree • Agree • Strongly Agree	Text
cms_partner_7	Answer to “My partner believes that in a conflicting situation both partners should move a step toward the other” Choices • Strongly Disagree • Disagree • Agree • Strongly Agree	One of • Strongly Disagree • Disagree • Agree • Strongly Agree	Text
Cope **(not a real column. this column is only providing information for cope_1 to cope_28)**	Prompt to question “These items deal with ways you've been coping with the stress in your life since you found out about the Coronavirus (COVID-19). There are many ways to try to deal with problems. These items ask what you've been doing to cope with this one. Obviously, different people deal with things in different ways, but we're interested in how you've tried to deal with it. Each item says something about a particular way of coping. We want to know to what extent you've been doing what the item says. How much or how frequently. Don't answer on the basis of whether it seems to be working or not–just whether or not y ou're doing it. USe these response choices. Try to rate each item separately in your mind from the others. Make your answers as true for you as you can” Choices • I haven't been doing this at all • I've been doing this a little bit • I've been doing this a medium amount • I've been doing this a lot	N/A	N/A
cope_1	Answer to “I've been turning to work or other activities to take my mind off things.” Choices • I haven't been doing this at all • I've been doing this a little bit • I've been doing this a medium amount • I've been doing this a lot	One of • I haven't been doing this at all • I've been doing this a little bit • I've been doing this a medium amount • I've been doing this a lot	Text
cope_2	Answer to “I've been concentrating my efforts on doing something about the situation I'm in.” Choices • I haven't been doing this at all • I've been doing this a little bit • I've been doing this a medium amount • I've been doing this a lot	One of • I haven't been doing this at all • I've been doing this a little bit • I've been doing this a medium amount • I've been doing this a lot	Text
cope_3	Answer to “I've been saying to myself “this isn't real”.” Choices • I haven't been doing this at all • I've been doing this a little bit • I've been doing this a medium amount • I've been doing this a lot	One of • I haven't been doing this at all • I've been doing this a little bit • I've been doing this a medium amount • I've been doing this a lot	Text
cope_4	Answer to “I've been using alcohol or other drugs to make myself feel better.” Choices • I haven't been doing this at all • I've been doing this a little bit • I've been doing this a medium amount • I've been doing this a lot **This field is only available upon request to protect participants’ privacy.**	One of • I haven't been doing this at all • I've been doing this a little bit • I've been doing this a medium amount • I've been doing this a lot	Text
cope_5	Answer to “I've been getting emotional support from others.” Choices • I haven't been doing this at all • I've been doing this a little bit • I've been doing this a medium amount • I've been doing this a lot	One of • I haven't been doing this at all • I've been doing this a little bit • I've been doing this a medium amount • I've been doing this a lot	Text
cope_6	Answer to “I've been trying to give you trying to deal with it.” Choices • I haven't been doing this at all • I've been doing this a little bit • I've been doing this a medium amount • I've been doing this a lot	One of • I haven't been doing this at all • I've been doing this a little bit • I've been doing this a medium amount • I've been doing this a lot	Text
cope_7	Answer to “I've been taking action to try to make the situation better.” Choices • I haven't been doing this at all • I've been doing this a little bit • I've been doing this a medium amount • I've been doing this a lot	One of • I haven't been doing this at all • I've been doing this a little bit • I've been doing this a medium amount • I've been doing this a lot	Text
cope_8	Answer to “I've been refusing to believe that it has happened.” Choices • I haven't been doing this at all • I've been doing this a little bit • I've been doing this a medium amount • I've been doing this a lot	One of • I haven't been doing this at all • I've been doing this a little bit • I've been doing this a medium amount • I've been doing this a lot	Text
cope_9	Answer to “I've been saying things to let my unpleasant feelings escape.” Choices • I haven't been doing this at all • I've been doing this a little bit • I've been doing this a medium amount • I've been doing this a lot	One of • I haven't been doing this at all • I've been doing this a little bit • I've been doing this a medium amount • I've been doing this a lot	Text
cope_10	Answer to “I've been getting help and advice from other people.” Choices • I haven't been doing this at all • I've been doing this a little bit • I've been doing this a medium amount • I've been doing this a lot	One of • I haven't been doing this at all • I've been doing this a little bit • I've been doing this a medium amount • I've been doing this a lot	Text
cope_11	Answer to “I've been using alcohol or other drugs to help me get through it.” Choices • I haven't been doing this at all • I've been doing this a little bit • I've been doing this a medium amount • I've been doing this a lot **This field is only available upon request to protect participants’ privacy.**	One of • I haven't been doing this at all • I've been doing this a little bit • I've been doing this a medium amount • I've been doing this a lot	Text
cope_12	Answer to “I've been trying to see it in a different light, to make it seem more positive.” Choices • I haven't been doing this at all • I've been doing this a little bit • I've been doing this a medium amount • I've been doing this a lot	One of • I haven't been doing this at all • I've been doing this a little bit • I've been doing this a medium amount • I've been doing this a lot	Text
cope_13	Answer to “I've been criticizing myself ” Choices • I haven't been doing this at all • I've been doing this a little bit • I've been doing this a medium amount • I've been doing this a lot	One of • I haven't been doing this at all • I've been doing this a little bit • I've been doing this a medium amount • I've been doing this a lot	Text
cope_14	Answer to “I've been trying to come up with a strategy about what to do.” Choices • I haven't been doing this at all • I've been doing this a little bit • I've been doing this a medium amount • I've been doing this a lot	One of • I haven't been doing this at all • I've been doing this a little bit • I've been doing this a medium amount • I've been doing this a lot	Text
cope_15	Answer to “I've been getting comfort and understanding from someone.” Choices • I haven't been doing this at all • I've been doing this a little bit • I've been doing this a medium amount • I've been doing this a lot	One of • I haven't been doing this at all • I've been doing this a little bit • I've been doing this a medium amount • I've been doing this a lot	
cope_16	Answer to “I've been giving up the attempt to cope.” Choices • I haven't been doing this at all • I've been doing this a little bit • I've been doing this a medium amount • I've been doing this a lot	One of • I haven't been doing this at all • I've been doing this a little bit • I've been doing this a medium amount • I've been doing this a lot	Text
cope_17	Answer to “I've been looking for something good in what is happening..” Choices • I haven't been doing this at all • I've been doing this a little bit • I've been doing this a medium amount • I've been doing this a lot	One of • I haven't been doing this at all • I've been doing this a little bit • I've been doing this a medium amount • I've been doing this a lot	Text
cope_18	Answer to “I've been making jokes about it.” Choices • I haven't been doing this at all • I've been doing this a little bit • I've been doing this a medium amount • I've been doing this a lot	One of • I haven't been doing this at all • I've been doing this a little bit • I've been doing this a medium amount • I've been doing this a lot	Text
cope_19	Answer to “I've been doing something to think about it less, such as going to movies, watching TV, reading, daydreaming, sleeping, or shopping.” Choices • I haven't been doing this at all • I've been doing this a little bit • I've been doing this a medium amount • I've been doing this a lot	One of • I haven't been doing this at all • I've been doing this a little bit • I've been doing this a medium amount • I've been doing this a lot	Text
cope_20	Answer to “I've been accepting the reality of the fact that it has happened.” Choices • I haven't been doing this at all • I've been doing this a little bit • I've been doing this a medium amount • I've been doing this a lot	One of • I haven't been doing this at all • I've been doing this a little bit • I've been doing this a medium amount • I've been doing this a lot	Text
cope_21	Answer to “I've been expressing my negative feelings.” Choices • I haven't been doing this at all • I've been doing this a little bit • I've been doing this a medium amount • I've been doing this a lot	One of • I haven't been doing this at all • I've been doing this a little bit • I've been doing this a medium amount • I've been doing this a lot	Text
cope_22	Answer to “I've been trying to find comfort in my religion or spiritual beliefs.” Choices • I haven't been doing this at all • I've been doing this a little bit • I've been doing this a medium amount • I've been doing this a lot	One of • I haven't been doing this at all • I've been doing this a little bit • I've been doing this a medium amount • I've been doing this a lot	Text
cope_23	Answer to “I've been trying to get advice or help from other people about what to do.” Choices • I haven't been doing this at all • I've been doing this a little bit • I've been doing this a medium amount • I've been doing this a lot	One of • I haven't been doing this at all • I've been doing this a little bit • I've been doing this a medium amount • I've been doing this a lot	Text
cope_24	Answer to “I've been learning to live with it.” Choices • I haven't been doing this at all • I've been doing this a little bit • I've been doing this a medium amount • I've been doing this a lot	One of • I haven't been doing this at all • I've been doing this a little bit • I've been doing this a medium amount • I've been doing this a lot	Text
cope_25	Answer to “I've been thinking hard about what steps to take.” Choices • I haven't been doing this at all • I've been doing this a little bit • I've been doing this a medium amount • I've been doing this a lot	One of • I haven't been doing this at all • I've been doing this a little bit • I've been doing this a medium amount • I've been doing this a lot	Text
cope_26	Answer to “I've been blaming myself for things that happened.” Choices • I haven't been doing this at all • I've been doing this a little bit • I've been doing this a medium amount • I've been doing this a lot	One of • I haven't been doing this at all • I've been doing this a little bit • I've been doing this a medium amount • I've been doing this a lot	Text
cope_27	Answer to “I've been praying or meditating.” Choices • I haven't been doing this at all • I've been doing this a little bit • I've been doing this a medium amount • I've been doing this a lot	One of • I haven't been doing this at all • I've been doing this a little bit • I've been doing this a medium amount • I've been doing this a lot	Text
cope_28	Answer to “I've been making fun of the situation” Choices • I haven't been doing this at all • I've been doing this a little bit • I've been doing this a medium amount • I've been doing this a lot	One of • I haven't been doing this at all • I've been doing this a little bit • I've been doing this a medium amount • I've been doing this a lot	Text
ecr **(not a real column. this column is only providing information for ecr_1 to ecr_12)**	Prompt to question “The following statements concern how you feel in romantic relationships. We are interested in how you generally experience relationships, not just in what is happening in a current relationship. Respond to each statement indicating how much you agree or disagree with it.” Choices • Strongly Disagree • Disagree • Slightly Disagree • Neutral • Slightly Agree • Agree • Strongly Agree	N/A	N/A
ecr_1	Answer to “It helps to turn to my romantic partner in times of need.” Choices • Strongly Disagree • Disagree • Slightly Disagree • Neutral • Slightly Agree • Agree • Strongly Agree	One of • Strongly Disagree • Disagree • Slightly Disagree • Neutral • Slightly Agree • Agree • Strongly Agree	Text
ecr_2	Answer to “I need a lot of reassurance that I am loved by my partner.” Choices • Strongly Disagree • Disagree • Slightly Disagree • Neutral • Slightly Agree • Agree • Strongly Agree	One of • Strongly Disagree • Disagree • Slightly Disagree • Neutral • Slightly Agree • Agree • Strongly Agree	Text
ecr_3	Answer to “I want to get close to my partner, but I keep pulling back.” Choices • Strongly Disagree • Disagree • Slightly Disagree • Neutral • Slightly Agree • Agree • Strongly Agree	One of • Strongly Disagree • Disagree • Slightly Disagree • Neutral • Slightly Agree • Agree • Strongly Agree	Text
ecr_4	Answer to “I find that my partner(s) don't want to get as close as I would like.” Choices • Strongly Disagree • Disagree • Slightly Disagree • Neutral • Slightly Agree • Agree • Strongly Agree	One of • Strongly Disagree • Disagree • Slightly Disagree • Neutral • Slightly Agree • Agree • Strongly Agree	Text
ecr_5	Answer to “I turn to my partner for many things, including comfort and reassurance.” Choices • Strongly Disagree • Disagree • Slightly Disagree • Neutral • Slightly Agree • Agree • Strongly Agree	One of • Strongly Disagree • Disagree • Slightly Disagree • Neutral • Slightly Agree • Agree • Strongly Agree	Text
ecr_6	Answer to “My desire to be very close sometimes scares people away.” Choices • Strongly Disagree • Disagree • Slightly Disagree • Neutral • Slightly Agree • Agree • Strongly Agree	One of • Strongly Disagree • Disagree • Slightly Disagree • Neutral • Slightly Agree • Agree • Strongly Agree	Text
ecr_7	Answer to “I try to avoid getting too close to my partner.” Choices • Strongly Disagree • Disagree • Slightly Disagree • Neutral • Slightly Agree • Agree • Strongly Agree	One of • Strongly Disagree • Disagree • Slightly Disagree • Neutral • Slightly Agree • Agree • Strongly Agree	Text
ecr_8	Answer to “I do not often worry about being abandoned.” Choices • Strongly Disagree • Disagree • Slightly Disagree • Neutral • Slightly Agree • Agree • Strongly Agree	One of • Strongly Disagree • Disagree • Slightly Disagree • Neutral • Slightly Agree • Agree • Strongly Agree	Text
ecr_9	Answer to “I usually discuss my problems and concerns with my partner.” Choices • Strongly Disagree • Disagree • Slightly Disagree • Neutral • Slightly Agree • Agree • Strongly Agree	One of • Strongly Disagree • Disagree • Slightly Disagree • Neutral • Slightly Agree • Agree • Strongly Agree	Text
ecr_10	Answer to “I get frustrated if romantic partners are not available if I need them.” Choices • Strongly Disagree • Disagree • Slightly Disagree • Neutral • Slightly Agree • Agree • Strongly Agree	One of • Strongly Disagree • Disagree • Slightly Disagree • Neutral • Slightly Agree • Agree • Strongly Agree	Text
ecr_11	Answer to “I am nervous when partners get too close to me.” Choices • Strongly Disagree • Disagree • Slightly Disagree • Neutral • Slightly Agree • Agree • Strongly Agree	One of • Strongly Disagree • Disagree • Slightly Disagree • Neutral • Slightly Agree • Agree • Strongly Agree	Text
ecr_12	Answer to “I worry that romantic partners won't care about me as much as I care about them.” Choices • Strongly Disagree • Disagree • Slightly Disagree • Neutral • Slightly Agree • Agree • Strongly Agree	One of • Strongly Disagree • Disagree • Slightly Disagree • Neutral • Slightly Agree • Agree • Strongly Agree	Text
ems **(not a real column. this column is only providing information for ems_1 to ems_19)**	Prompt to question “Please report the extent to which the following statements describe how you are controlling (that is, regulate and manage) your emotions related to the coronavirus (COVID-19) and the situation surrounding it.” Choices • 1 (Not at all) • 2 • 3 • 4 • 5 • 6 • 7 (Extremely)	N/A	N/A
ems_1	Answer to “To manage my feelings about the coronavirus (COVID-19) and the situation surrounding it, I am reinterpreting the meaning of the situation in more neutral, less negative terms.” Choices • 1 (Not at all) • 2 • 3 • 4 • 5 • 6 • 7 (Extremely)	1-7	Number
ems_2	Answer to “To feel more positive about the coronavirus (COVID-19) and the situation surrounding it, I am changing the way I'm thinking about the situation.” Choices • 1 (Not at all) • 2 • 3 • 4 • 5 • 6 • 7 (Extremely)	1-7	Number
ems_3	Answer to “To deal with my emotions related to the coronavirus (COVID-19) and the situation surrounding it, I am thinking about the situation in a way that helps me stay calm.” Choices • 1 (Not at all) • 2 • 3 • 4 • 5 • 6 • 7 (Extremely)	1-7	Number
ems_4	Answer to “How much control do you feel you have over the coronavirus (COVID-19).” Choices • 1 (No control at all) • 2 • 3 • 4 (A moderate amount of control) • 5 • 6 • 7 (Complete control)	1-7	Number
ems_5	Answer to “How much control do you feel you have to protect yourself from getting coronavirus (COVID-19).” Choices • 1 (No control at all) • 2 • 3 • 4 (A moderate amount of control) • 5 • 6 • 7 (Complete control)	1-7	Number
ems_6	Answer to “How much control do you feel you have over preventing coronavirus (COVID-19) from spreading.” Choices • 1 (No control at all) • 2 • 3 • 4 (A moderate amount of control) • 5 • 6 • 7 (Complete control)	1-7	Number
ems_7	Answer to “How much control do you feel you have to protect your family from getting coronavirus (COVID-19).” Choices • 1 (No control at all) • 2 • 3 • 4 (A moderate amount of control) • 5 • 6 • 7 (Complete control)	1-7	Number
ems_9	Answer to “I am worried about the coronavirus (COVID-19) and the situation surrounding it.” Choices • 1 (Not at all) • 2 • 3 • 4 • 5 • 6 • 7 (Extremely)	1-7	Number
ems_10	Answer to “I am anxious about the coronavirus (COVID-19) and the situation surrounding it.” Choices • 1 (Not at all) • 2 • 3 • 4 • 5 • 6 • 7 (Extremely)	1-7	Number
ems_11	Answer to “It is difficult to focus on things other than the coronavirus (COVID-19) and the situation surrounding it.” Choices • 1 (Not at all) • 2 • 3 • 4 • 5 • 6 7 (Extremely)	1-7	Number
ems_12	Answer to “I am stressed about the coronavirus (COVID-19) and the situation surrounding it.” Choices • 1 (Not at all) • 2 • 3 • 4 • 5 • 6 • 7 (Extremely)	1-7	Number
ems_13	Answer to “I feel depressed about the coronavirus (COVID-19) and the situation surrounding it.” Choices • 1 (Not at all) • 2 • 3 • 4 • 5 • 6 • 7 (Extremely)	1-7	Number
ems_14	Answer to “I feel sad about the coronavirus (COVID-19) and the situation surrounding it.” Choices • 1 (Not at all) • 2 • 3 • 4 • 5 • 6 • 7 (Extremely)	1-7	Number
ems_15	Answer to “I am unhappy because of the coronavirus (COVID-19) and the situation surrounding it.” Choices • 1 (Not at all) • 2 • 3 • 4 • 5 • 6 • 7 (Extremely)	1-7	Number
ems_16	Answer to “Everyone can learn to control their feelings about the coronavirus (COVID-19).” Choices • 1 (Not at all) • 2 • 3 • 4 • 5 • 6 • 7 (Extremely)	1-7	Number
ems_17	Answer to “People can change their feelings if they want to about the coronavirus (COVID-19).” Choices • 1 (Not at all) • 2 • 3 • 4 • 5 • 6 • 7 (Extremely)	1-7	Number
ems_18	Answer to “No matter how hard they try, people can't really change their feelings about the coronavirus (COVID-19).” Choices • 1 (Not at all) • 2 • 3 • 4 • 5 • 6 • 7 (Extremely)	1-7	Number
ems_19	Answer to “People have very little control over their feelings about the coronavirus (COVID-19).” Choices • 1 (Not at all) • 2 • 3 • 4 • 5 • 6 • 7 (Extremely)	1-7	Number
exposure_1	Answer to “Have you been diagnosed with the coronavirus.” Choices • Yes • No	One of • YES • NO	Text
exposure_2	Answer to “Have you been hospitalised for the coronavirus.” Choices • Yes • No	One of • YES • NO	Text
exposure_3	Answer to “Have you been quarantined for the coronavirus.” Choices • Yes • No	One of • YES • NO	Text
exposure_4	Answer to “Have you come in contact with someone who has been in a high risk area for the coronavirus.” Choices • Yes • No	One of • YES • NO	Text
exposure_5	Answer to “Have you come in contact with someone who has a possible or confirmed case of the coronavirus.” Choices • Yes • No	One of • YES • NO	Text
exposure_6	Answer to “Has anyone you know personally (friend, relative, partner) had a possible or confirmed case of the coronavirus.” Choices • Yes • No	One of • YES • NO	Text
exposure_7	Answer to “Are you immunocompromised or have other health conditions that would make you at higher risk for the coronavirus.” Choices • Yes • No	One of • YES • NO	Text
fim_1	Answer to “Personicle seems implementable.” Choices: • Completely Disagree • Disagree • Neither Agree Nor Disagree • Agree • Completely Agree	One of: • Completely Disagree • Disagree • Neither Agree Nor Disagree • Agree • Completely Agree	Text
fim_2	Answer to “Personicle seems possible.” Choices: • Completely Disagree • Disagree • Neither Agree Nor Disagree • Agree • Completely Agree	One of: • Completely Disagree • Disagree • Neither Agree Nor Disagree • Agree • Completely Agree	Text
fim_3	Answer to “Personicle seems doable.” Choices: • Completely Disagree • Disagree • Neither Agree Nor Disagree • Agree • Completely Agree	One of: • Completely Disagree • Disagree • Neither Agree Nor Disagree • Agree • Completely Agree	Text
fim_4	Answer to “Personicle seems easy to use.” Choices: • Completely Disagree • Disagree • Neither Agree Nor Disagree • Agree • Completely Agree	One of: • Completely Disagree • Disagree • Neither Agree Nor Disagree • Agree • Completely Agree	Text
gad_1	Answer to “Feeling nervous, anxious, or on edge” • 0 - Not at all • 1 - Several Days • 2 - More than half the days • 3 - Nearly every day	0-3	Number
gad_2	Answer to “Not being able to stop or control anything” • 0 - Not at all • 1 - Several Days • 2 - More than half the days • 3 - Nearly every day	0-3	Number
gad_3	Answer to “Worrying too much about different things” • 0 - Not at all • 1 - Several Days • 2 - More than half the days • 3 - Nearly every day	0-3	Number
gad_4	Answer to “Trouble relaxing” • 0 - Not at all • 1 - Several Days • 2 - More than half the days • 3 - Nearly every day	0-3	Number
gad_5	Answer to “Being so restless that it is hard to sit still” • 0 - Not at all • 1 - Several Days • 2 - More than half the days • 3 - Nearly every day	0-3	Number
gad_6	Answer to “Becoming easily annoyed or irritable” • 0 - Not at all • 1 - Several Days • 2 - More than half the days • 3 - Nearly every day	0-3	Number
gad_7	Answer to “Feeling afraid as if something awful might happen” • 0 - Not at all • 1 - Several Days • 2 - More than half the days • 3 - Nearly every day	0-3	Number
ha_1	Answer to “How stressed/anxious are you about your health in general?” Choices: • Not at all stressed/anxious • A little stressed/anxious • Moderately stressed/anxious • Very stressed/anxious • Extremely stressed/anxious	One of: • Not at all stressed/anxious • A little stressed/anxious • Moderately stressed/anxious • Very stressed/anxious • Extremely stressed/anxious	Text
ha_2	Answer to “How stressed/anxious are you about getting the coronavirus?” Choices: • Not at all stressed/anxious • A little stressed/anxious • Moderately stressed/anxious • Very stressed/anxious • Extremely stressed/anxious	One of: • Not at all stressed/anxious • A little stressed/anxious • Moderately stressed/anxious • Very stressed/anxious • Extremely stressed/anxious	Text
hm_goals	Answer to “Pick the mantra that best describes you when it comes to the coronavirus” Choices: • Everyone chill out • Keep calm and carry on • Keep calm and carry hand sanitizer • Better safe than sorry • It's the end of the world as we know it • It's the end of the world as we know it, but I feel fine • Cancel everything • There are bigger fish to fry (more important problems need our attention)	One of: • Everyone chill out • Keep calm and carry on • Keep calm and carry hand sanitizer • Better safe than sorry • It's the end of the world as we know it • It's the end of the world as we know it, but I feel fine • Cancel everything • There are bigger fish to fry (more important problems need our attention)	Text
hm_body_1	Answer to “In general, my body has remarkable self-healing properties and can heal itself from many illnesses.” Choices: • Strongly disagree • Disagree • Somewhat disagree • Somewhat agree • Agree • Strongly agree	One of: • Strongly disagree • Disagree • Somewhat disagree • Somewhat agree • Agree • Strongly agree	Text
hm_body_2	Answer to “In general, my body is capable of handling an illness like the coronavirus” Choices: • Strongly disagree • Disagree • Somewhat disagree • Somewhat agree • Agree • Strongly agree	One of: • Strongly disagree • Disagree • Somewhat disagree • Somewhat agree • Agree • Strongly agree	Text
hm_body_3	Answer to “Getting sick from the coronavirus means that my body has failed or betrayed me” Choices: • Strongly disagree • Disagree • Somewhat disagree • Somewhat agree • Agree • Strongly agree	One of: • Strongly disagree • Disagree • Somewhat disagree • Somewhat agree • Agree • Strongly agree	Text
hm_ill_1	Answer to “The coronavirus outbreak can be managed so that people in our society can live life as normal.” Choices: • Strongly disagree • Disagree • Somewhat disagree • Somewhat agree • Agree • Strongly agree	One of: • Strongly disagree • Disagree • Somewhat disagree • Somewhat agree • Agree • Strongly agree	Text
hm_ill_2	Answer to “The coronavirus outbreak is a global catastrophe that is wreaking havoc on our society” Choices: • Strongly disagree • Disagree • Somewhat disagree • Somewhat agree • Agree • Strongly agree	One of: • Strongly disagree • Disagree • Somewhat disagree • Somewhat agree • Agree • Strongly agree	Text
hm_ill_3	Answer to “The coronavirus outbreak can be an opportunity for our society to make positive changes.” Choices: • Strongly disagree • Disagree • Somewhat disagree • Somewhat agree • Agree • Strongly agree	One of: • Strongly disagree • Disagree • Somewhat disagree • Somewhat agree • Agree • Strongly agree	Text
hm_ill_4	Answer to “Having the coronavirus ruins or spoils most parts of a person's life.” Choices: • Strongly disagree • Disagree • Somewhat disagree • Somewhat agree • Agree • Strongly agree	One of: • Strongly disagree • Disagree • Somewhat disagree • Somewhat agree • Agree • Strongly agree	Text
hm_ill_5	Answer to “The coronavirus can be managed so it's not too disruptive to a person's life.” Choices: • Strongly disagree • Disagree • Somewhat disagree • Somewhat agree • Agree • Strongly agree	One of: • Strongly disagree • Disagree • Somewhat disagree • Somewhat agree • Agree • Strongly agree	Text
hm_ill_6	Answer to “Getting the coronavirus can be an opportunity for a person to make positive life changes” Choices: • Strongly disagree • Disagree • Somewhat disagree • Somewhat agree • Agree • Strongly agree	One of: • Strongly disagree • Disagree • Somewhat disagree • Somewhat agree • Agree • Strongly agree	Text
hm_social **(not a real column. this column is only providing information for hm_social_1 to hm_social_3)**	Prompt to question “Please click on the area in the picture that best aligns with your personal opinion on the effect of changes in an individual's physical health”Image: Graph of eight circles with each consecutive circles within each other; from the most inner circle to outer: • That person • Their Family • Their friends • Their coworkers, colleagues, and peers • Their neighborhood • Their community • Their world		N/A
hm_social_1_1_x	X location of the participant choice on the image for the prompt: “A decline in a person's physical health will affect:”	0-783	Number
hm_social_1_1_y	Y location of the participant choice on the image for the prompt: “A decline in a person's physical health will affect:”	0-618	Number
hm_social_1_choice	Their choice on the image.One of: • Person: That person • Family: Their Family • friends: Their friends • peers: Their coworkers, colleagues, and peers • Neighborhood: Their neighborhood • Community: Their community • The world: Their world	One of: • Person • Family • friends • peers • Neighborhood • Community • country • The world	Text
hm_social_2_1_x	X location of the participant choice on the image for the prompt: “An improvement in a person's physical health will affect:”	0-783	Number
hm_social_2_1_y	Y location of the participant choice on the image for the prompt: “An improvement in a person's physical health will affect:”	0-618	Number
hm_social_2_choice	Their choice on the image.One of: • Person: That person • Family: Their Family • friends: Their friends • peers: Their coworkers, colleagues, and peers • Neighborhood: Their neighborhood • Community: Their community • The world: Their world	One of: • Person • Family • friends • peers • Neighborhood • Community • country • The world	Text
hm_social_3_1_x	X location of the participant choice on the image for the prompt: “An improvement in a person's physical health is a sign of strength for:”	0-783	Number
hm_social_3_1_y	Y location of the participant choice on the image for the prompt: “An improvement in a person's physical health is a sign of strength for:”	0-618	Number
hm_social_3_choice	Their choice on the image.One of: • Person: That person • Family: Their Family • friends: Their friends • peers: Their coworkers, colleagues, and peers • Neighborhood: Their neighborhood • Community: Their community • The world: Their world	One of: • Person • Family • friends • peers • Neighborhood • Community • country • The world	Text
hpc_1	Answer to “Your health in general?” Choices: • 1 - No control • 2 - Slight control • 3 - Some control • 4 - A lot of control	1-4	Number
hpc_2	Answer to “Whether or not you contract the coronavirus?” Choices: • 1 - No control • 2 - Slight control • 3 - Some control • 4 - A lot of control	1-4	Number
hpc_3	Answer to “Your experience (e.g. symptoms and treatment) of the coronavirus, if you do get it?” Choices: • 1 - No control • 2 - Slight control • 3 - Some control • 4 - A lot of control	1-4	Number
hpt_1	Answer to “I don't think I could get COVID-19” Choices: • 1 - not at all • 2 • 3 • 4 - a great deal	1-4	Number
hpt_2	Answer to “I feel nervous about getting COVID-19” Choices: • 1 - not at all • 2 • 3 • 4 - a great deal	1-4	Number
hpt_3	Answer to “COVID-19 is threatening my health” Choices: • 1 - not at all • 2 • 3 • 4 - a great deal	1-4	Number
hpt_4	Answer to “I don't feel worried about getting COVID-19” Choices: • 1 - not at all • 2 • 3 • 4 - a great deal	1-4	Number
hpt_5	Answer to “My daily routine has been disrupted due to thoughts about COVID-19” Choices: • 1 - not at all • 2 • 3 • 4 - a great deal	1-4	Number
iam_1	Answer to “Personicle seems fitting for my work.” Choices: • Completely Disagree • Disagree • Neither Agree Nor Disagree • Agree • Completely Agree	One of: • Completely Disagree • Disagree • Neither Agree Nor Disagree • Agree • Completely Agree	Text
iam_2	Answer to “Personicle seems suitable for my work.” Choices: • Completely Disagree • Disagree • Neither Agree Nor Disagree • Agree • Completely Agree	One of: • Completely Disagree • Disagree • Neither Agree Nor Disagree • Agree • Completely Agree	Text
iam_3	Answer to “Personicle seems applicable for my work.” Choices: • Completely Disagree • Disagree • Neither Agree Nor Disagree • Agree • Completely Agree	One of: • Completely Disagree • Disagree • Neither Agree Nor Disagree • Agree • Completely Agree	Text
iam_4	Answer to “Personicle seems like a good match for my work.” Choices: • Completely Disagree • Disagree • Neither Agree Nor Disagree • Agree • Completely Agree	One of: • Completely Disagree • Disagree • Neither Agree Nor Disagree • Agree • Completely Agree	Text
ims_1	Answer to “My partner fulfills my need for intimacy (sharing personal thoughts, secrets, etc.)” Choices: • Don't Agree at All • Agree Slightly • Agree Moderately • Agree Completely	One of: • Don't Agree at All • Agree Slightly • Agree Moderately • Agree Completely	Text
ims_2	Answer to “My partner fulfills my need for companionship (doing things together, enjoying each other's company, etc.)” Choices: • Don't Agree at All • Agree Slightly • Agree Moderately • Agree Completely	One of: • Don't Agree at All • Agree Slightly • Agree Moderately • Agree Completely	Text
ims_3	Answer to “My partner fulfills my sexual needs (holding hands, kissing, etc.)” Choices: • Don't Agree at All • Agree Slightly • Agree Moderately • Agree Completely	One of: • Don't Agree at All • Agree Slightly • Agree Moderately • Agree Completely	Text
ims_4	Answer to “My partner fulfills my needs for security (feeling trusting, comfortable in a stable relationship, etc.)” Choices: • Don't Agree at All • Agree Slightly • Agree Moderately • Agree Completely	One of: • Don't Agree at All • Agree Slightly • Agree Moderately • Agree Completely	Text
ims_5	Answer to “My partner fulfills my needs for emotional involvement (feeling emotionally attached, feeling good when another feels good, etc.)” Choices: • Don't Agree at All • Agree Slightly • Agree Moderately • Agree Completely	One of: • Don't Agree at All • Agree Slightly • Agree Moderately • Agree Completely	Text
ims_6	Answer to “I feel satisfied with our relationship” Choices: • Don't Agree at All • 1 • 2 • 3 • Agree Somewhat • 5 • 6 • 7 • Agree Completely	One of: • Don't Agree at All • 1 • 2 • 3 • Agree Somewhat • 5 • 6 • 7 • Agree Completely	Text/Number
ims_7	Answer to “My relationship is much better than others’ relationships” Choices: • Don't Agree at All • 1 • 2 • 3 • Agree Somewhat • 5 • 6 • 7 • Agree Completely	One of: • Don't Agree at All • 1 • 2 • 3 • Agree Somewhat • 5 • 6 • 7 • Agree Completely	Text/Number
ims_8	Answer to “My relationship is close to ideal” Choices: • Don't Agree at All • 1 • 2 • 3 • Agree Somewhat • 5 • 6 • 7 • Agree Completely	One of: • Don't Agree at All • 1 • 2 • 3 • Agree Somewhat • 5 • 6 • 7 • Agree Completely	Text/Number
ims_9	Answer to “Our relationship makes me very happy” Choices: • Don't Agree at All • 1 • 2 • 3 • Agree Somewhat • 5 • 6 • 7 • Agree Completely	One of: • Don't Agree at All • 1 • 2 • 3 • Agree Somewhat • 5 • 6 • 7 • Agree Completely	Text/Number
ims_10	Answer to “Our relationship does a good job of fulfilling my needs for intimacy, companionship, etc.” Choices: • Don't Agree at All • 1 • 2 • 3 • Agree Somewhat • 5 • 6 • 7 • Agree Completely	One of: • Don't Agree at All • 1 • 2 • 3 • Agree Somewhat • 5 • 6 • 7 • Agree Completely	Text/Number
ims_11	Answer to “My needs for intimacy (sharing personal thoughts, secrets, etc.) could be fulfilled in alternative relationships.” Choices: • Don't Agree at All • Agree Slightly • Agree Moderately • Agree Completely	One of: • Don't Agree at All • Agree Slightly • Agree Moderately • Agree Completely	Text
ims_12	Answer to “My needs for companionship (doing things together, enjoying each other's company, etc.) could be fulfilled in alternative relationships.” Choices: • Don't Agree at All • Agree Slightly • Agree Moderately • Agree Completely	One of: • Don't Agree at All • Agree Slightly • Agree Moderately • Agree Completely	Text
ims_13	Answer to question “My sexual needs (holding hands, kissing, etc.) could be fulfilled in alternative relationships.” Choices: • Don't Agree at All • Agree Slightly • Agree Moderately • Agree Completely	One of: • Don't Agree at All • Agree Slightly • Agree Moderately • Agree Completely	Text
ims_14	Answer to question “My needs for security (feeling trusting, comfortable in a stable relationship, etc.) could be fulfilled in alternative relationships.” Choices: • Don't Agree at All • Agree Slightly • Agree Moderately • Agree Completely	One of: • Don't Agree at All • Agree Slightly • Agree Moderately • Agree Completely	Text
ims_15	Answer to question “My needs for emotional involvement (feeling emotionally attached, feeling good when another feels good, etc.) could be fulfilled in alternative relationships.” Choices: • Don't Agree at All • Agree Slightly • Agree Moderately • Agree Completely	One of: • Don't Agree at All • Agree Slightly • Agree Moderately • Agree Completely	Text
ims_16	Answer to question “Our relationship does a good job of fulfilling my needs for intimacy, companionship, etc.” Choices: • Don't Agree at All • 1 • 2 • 3 • Agree Somewhat • 5 • 6 • 7 • Agree Completely	One of: • Don't Agree at All • 1 • 2 • 3 • Agree Somewhat • 5 • 6 • 7 • Agree Completely	Text/Number
ims_17	Answer to question “Our relationship does a good job of fulfilling my needs for intimacy, companionship, etc.” Choices: • Don't Agree at All • 1 • 2 • 3 • Agree Somewhat • 5 • 6 • 7 • Agree Completely	One of: • Don't Agree at All • 1 • 2 • 3 • Agree Somewhat • 5 • 6 • 7 • Agree Completely	Text/Number
ims_18	Answer to question “Our relationship does a good job of fulfilling my needs for intimacy, companionship, etc.” Choices: • Don't Agree at All • 1 • 2 • 3 • Agree Somewhat • 5 • 6 • 7 • Agree Completely	One of: • Don't Agree at All • 1 • 2 • 3 • Agree Somewhat • 5 • 6 • 7 • Agree Completely	Text/Number
ims_19	Answer to question “Our relationship does a good job of fulfilling my needs for intimacy, companionship, etc.” Choices: • Don't Agree at All • 1 • 2 • 3 • Agree Somewhat • 5 • 6 • 7 • Agree Completely	One of: • Don't Agree at All • 1 • 2 • 3 • Agree Somewhat • 5 • 6 • 7 • Agree Completely	Text/Number
ims_20	Answer to question “Our relationship does a good job of fulfilling my needs for intimacy, companionship, etc.” Choices: • Don't Agree at All • 1 • 2 • 3 • Agree Somewhat • 5 • 6 • 7 • Agree Completely	One of: • Don't Agree at All • 1 • 2 • 3 • Agree Somewhat • 5 • 6 • 7 • Agree Completely	Text/Number
ims_21	Answer to question “I have invested a great deal of time in our relationship.” Choices: • Don't Agree at All • Agree Slightly • Agree Moderately • Agree Completely	One of: • Don't Agree at All • Agree Slightly • Agree Moderately • Agree Completely	Text
ims_22	Answer to question “I have told my partner many private things about myself (I disclose secrets to him/her).” Choices: • Don't Agree at All • Agree Slightly • Agree Moderately • Agree Completely	One of: • Don't Agree at All • Agree Slightly • Agree Moderately • Agree Completely	Text
ims_23	Answer to question “My partner and I have an intellectual life together that would be difficult to replace.” Choices: • Don't Agree at All • Agree Slightly • Agree Moderately • Agree Completely	One of: • Don't Agree at All • Agree Slightly • Agree Moderately • Agree Completely	Text
ims_24	Answer to question “My sense of personal identity (who I am) is linked to my partner and our relationship.” Choices: • Don't Agree at All • Agree Slightly • Agree Moderately • Agree Completely	One of: • Don't Agree at All • Agree Slightly • Agree Moderately • Agree Completely	Text
ims_25	Answer to question “My partner and I share many memories.” Choices: • Don't Agree at All • Agree Slightly • Agree Moderately • Agree Completely	One of: • Don't Agree at All • Agree Slightly • Agree Moderately • Agree Completely	Text
ims_26	Answer to question “I have put a great deal into our relationship that I would lose if the relationship were to end. ” Choices: • Don't Agree at All • 1 • 2 • 3 • Agree Somewhat • 5 • 6 • 7 • Agree Completely	One of: • Don't Agree at All • 1 • 2 • 3 • Agree Somewhat • 5 • 6 • 7 • Agree Completely	Text/Number
ims_27	Answer to question “Many aspects of my life have become linked to my partner (recreational activities, etc.) and I would lose all of this if we were to break up.” Choices: • Don't Agree at All • 1 • 2 • 3 • Agree Somewhat • 5 • 6 • 7 • Agree Completely	One of: • Don't Agree at All • 1 • 2 • 3 • Agree Somewhat • 5 • 6 • 7 • Agree Completely	Text/Number
ims_28	Answer to question “I feel very involved in our relationship like I have put a great deal into it. ” Choices: • Don't Agree at All • 1 • 2 • 3 • Agree Somewhat • 5 • 6 • 7 • Agree Completely	One of: • Don't Agree at All • 1 • 2 • 3 • Agree Somewhat • 5 • 6 • 7 • Agree Completely	Text/Number
ims_29	Answer to question “My relationships with friends and family members would be complicated if my partner and I were to break up (e.g. partner is friends with people I care about).Choices: • Don't Agree at All • 1 • 2 • 3 • Agree Somewhat • 5 • 6 • 7 • Agree Completely	One of: • Don't Agree at All • 1 • 2 • 3 • Agree Somewhat • 5 • 6 • 7 • Agree Completely	Text/Number
ims_30	Answer to question “Compared to other people I know, I have invested a great deal in my relationship with my partner.” Choices: • Don't Agree at All • 1 • 2 • 3 • Agree Somewhat • 5 • 6 • 7 • Agree Completely	One of: • Don't Agree at All • 1 • 2 • 3 • Agree Somewhat • 5 • 6 • 7 • Agree Completely	Text/Number
ims_31	Answer to question “I am committed to maintaining my relationship with my partner.” Choices: • Don't Agree at All • 1 • 2 • 3 • Agree Somewhat • 5 • 6 • 7 • Agree Completely	One of: • Don't Agree at All • 1 • 2 • 3 • Agree Somewhat • 5 • 6 • 7 • Agree Completely	Text/Number
ims_32	Answer to question “I want our relationship to last for a very long time.” Choices: • Don't Agree at All • 1 • 2 • 3 • Agree Somewhat • 5 • 6 • 7 • Agree Completely	One of: • Don't Agree at All • 1 • 2 • 3 • Agree Somewhat • 5 6• • 7 • Agree Completely	Text/Number
ims_33	Answer to question “I feel very attached to our relationship - very strongly linked to my partner.” Choices: • Don't Agree at All • 1 • 2 • 3 • Agree Somewhat • 5 • 6 • 7 • Agree Completely	One of: • Don't Agree at All • 1 • 2 • 3 • Agree Somewhat • 5 • 6 • 7 • Agree Completely	Text/Number
ims_34	Answer to question “It is likely that I will date someone other than my partner within the next year.” Choices: • Don't Agree at All • 1 • 2 • 3 • Agree Somewhat • 5 • 6 • 7 • Agree Completely	One of: • Don't Agree at All • 1 • 2 • 3 • Agree Somewhat • 5 • 6 • 7 • Agree Completely	Text/Number
ims_35	Answer to question “I would not feel very upset if our relationship were to end in the near future.” Choices: • Don't Agree at All • 1 • 2 • 3 • Agree Somewhat • 5 • 6 • 7 • Agree Completely	One of: • Don't Agree at All • 1 • 2 • 3 • Agree Somewhat • 5 • 6 • 7 • Agree Completely	Text/Number
ims_36	Answer to question “I want our relationship to last forever.” Choices: • Don't Agree at All • 1 • 2 • 3 • Agree Somewhat • 5 • 6 • 7 • Agree Completely	One of: • Don't Agree at All • 1 • 2 • 3 • Agree Somewhat • 5 • 6 • 7 • Agree Completely	Text/Number
ims_37	Answer to question “I am oriented toward the long-term future of my relationship (for example, I imagine being with my partner several years from now).” Choices: • Don't Agree at All • 1 • 2 • 3 • Agree Somewhat • 5 • 6 • 7 • Agree Completely	One of: • Don't Agree at All • 1 • 2 • 3 • Agree Somewhat • 5 • 6 • 7 • Agree Completely	Text/Number
ios	Prompt to question “Please select the picture below that best represents your current relationship with your romantic partner”		Text
ios_pic	Prompt to question “Please select the picture below that best represents your current relationship with your romantic partner” Choices: • Self and Other separate • Self and Other begin intersecting • Self and Other intersect 25% • Self and Other intersect > than previous • Self and Other intersect approx. 50% • Self and Other intersect > 50% • Self and Other intersecting almost 100% • Not Applicable	One of	Diagrams
isolation_1	Answer to question “In the last week, have you felt socially isolated?” Choices: • Yes • No	One of: • Yes • No	Text
isolation_2	Answer to question “In the last week, have you been socially distancing yourself from others (minimizing your in-person interactions)?” Choices: • Yes • No	One of: • Yes • No	Text
isolation_3	Answer to question “In the last week, have you quarantined yourself from others (not left your home unless absolutely necessary)?” Choices: • Yes • No	One of: • Yes • No	Text
isolation_4a	Answer to question “Compared to before the coronavirus, how much time do you spend interacting with people in person?” Choices: • 1 - Much Less • 2 • 3 - About the Same • 4 • 5 - Much More	One of: • 1 - Much Less • 2 • 3 - About the Same • 4 • 5 - Much More	Text
isolation_4b	Answer to question “What is the quality of those interactions?” Choices: • 1 - Very Poor • 2 • 3 - Neutral • 4 • 5 - Very Good	One of: • 1 - Very Poor • 2 • 3 - Neutral • 4 • 5 - Very Good	Text
isolation_5	Answer to question “Compared to before the coronavirus, how has the quality of those interactions changed?” Choices: • 1 - Worse • 2 • 3 - About the Same • 4 • 5 - Much Better	One of: • 1 - Worse • 2 • 3 - About the Same • 4 • 5 - Much Better	Text
isolation_6a	Answer to question “Compared to before the coronavirus, how much time do you spend interacting with people **online** or **over the phone**?” Choices: • 1 - Much Less • 2 • 3 - About the Same • 4 • 5 - Much More	One of: • 1 - Much Less • 2 • 3 - About the Same • 4 • 5 - Much More	Text
isolation_6b	Answer to question “What is the quality of those interactions?” Choices: • 1 - Very Poor • 2 • 3 - Neutral • 4 • 5 - Very Good	One of: • 1 - Very Poor • 2 • 3 - Neutral • 4 • 5 - Very Good	Text
isolation_7	Answer to question “Compared to before the coronavirus, how has the quality of those interactions changed?” Choices: • 1 - Much Worse • 2 • 3 - About the Same • 4 • 5 - Much Better	One of: • 1 - Much Worse • 2 • 3 - About the Same • 4 • 5 - Much Better	Text
mspss_1	Answer to “There is a special person who is around when I am in need.” Choices: • Very Strongly Disagree • Strongly Disagree • Mildly Disagree • Neutral • Mildly Agree • Strongly Agree • Very Strongly Agree	One of: • Very Strongly Disagree • Strongly Disagree • Mildly Disagree • Neutral • Mildly Agree • Strongly Agree • Very Strongly Agree	Text
mspss_2	Answer to “There is a special person whom I can share my joys and sorrows.” Choices: • Very Strongly Disagree • Strongly Disagree • Mildly Disagree • Neutral • Mildly Agree • Strongly Agree • Very Strongly Agree	One of: • Very Strongly Disagree • Strongly Disagree • Mildly Disagree • Neutral • Mildly Agree • Strongly Agree • Very Strongly Agree	Text
mspss_3	Answer to “My family really tries to help me” Choices: • Very Strongly Disagree • Strongly Disagree • Mildly Disagree • Neutral • Mildly Agree • Strongly Agree • Very Strongly Agree	One of: • Very Strongly Disagree • Strongly Disagree • Mildly Disagree • Neutral • Mildly Agree • Strongly Agree • Very Strongly Agree	Text
mspss_4	Answer to “I get the emotional help and support I need from my family” Choices: • Very Strongly Disagree • Strongly Disagree • Mildly Disagree • Neutral • Mildly Agree • Strongly Agree • Very Strongly Agree	One of: • Very Strongly Disagree • Strongly Disagree • Mildly Disagree • Neutral • Mildly Agree • Strongly Agree • Very Strongly Agree	Text
mspss_5	Answer to “I have a special person who is a real source of comfort to me.” Choices: • Very Strongly Disagree • Strongly Disagree • Mildly Disagree • Neutral • Mildly Agree • Strongly Agree • Very Strongly Agree	One of: • Very Strongly Disagree • Strongly Disagree • Mildly Disagree • Neutral • Mildly Agree • Strongly Agree • Very Strongly Agree	Text
mspss_6	Answer to “My friends really try to help me.” Choices: • Very Strongly Disagree • Strongly Disagree • Mildly Disagree • Neutral • Mildly Agree • Strongly Agree • Very Strongly Agree	One of: • Very Strongly Disagree • Strongly Disagree • Mildly Disagree • Neutral • Mildly Agree • Strongly Agree • Very Strongly Agree	Text
mspss_7	Answer to “I can count on my friends when things go wrong” Choices: • Very Strongly Disagree • Strongly Disagree • Mildly Disagree • Neutral • Mildly Agree • Strongly Agree • Very Strongly Agree	One of: • Very Strongly Disagree • Strongly Disagree • Mildly Disagree • Neutral • Mildly Agree • Strongly Agree • Very Strongly Agree	Text
mspss_8	Answer to “I can talk about my problems with my family” Choices: • Very Strongly Disagree • Strongly Disagree • Mildly Disagree • Neutral • Mildly Agree • Strongly Agree • Very Strongly Agree	One of: • Very Strongly Disagree • Strongly Disagree • Mildly Disagree • Neutral • Mildly Agree • Strongly Agree • Very Strongly Agree	Text
mspss_9	Answer to “I have friends with whom I can share my joys and sorrows.” Choices: • Very Strongly Disagree • Strongly Disagree • Mildly Disagree • Neutral • Mildly Agree • Strongly Agree • Very Strongly Agree	One of: • Very Strongly Disagree • Strongly Disagree • Mildly Disagree • Neutral • Mildly Agree • Strongly Agree • Very Strongly Agree	Text
mspss_10	Answer to “There is a special person in my life who cares about my feelings.” Choices: • Very Strongly Disagree • Strongly Disagree • Mildly Disagree • Neutral • Mildly Agree • Strongly Agree • Very Strongly Agree	One of: • Very Strongly Disagree • Strongly Disagree • Mildly Disagree • Neutral • Mildly Agree • Strongly Agree • Very Strongly Agree	Text
mspss_11	Answer to “My family is willing to help me make decisions” Choices: • Very Strongly Disagree • Strongly Disagree • Mildly Disagree • Neutral • Mildly Agree • Strongly Agree • Very Strongly Agree	One of: • Very Strongly Disagree • Strongly Disagree • Mildly Disagree • Neutral • Mildly Agree • Strongly Agree • Very Strongly Agree	Text
mspss_12	Answer to “I can talk about my problems with my friends” Choices: • Very Strongly Disagree • Strongly Disagree • Mildly Disagree • Neutral • Mildly Agree • Strongly Agree • Very Strongly Agree	One of: • Very Strongly Disagree • Strongly Disagree • Mildly Disagree • Neutral • Mildly Agree • Strongly Agree • Very Strongly Agree	Text
panas_1	Answer to question “Interested” Choices: • Very Slightly or Not at All • A little • Moderately • Quite a bit • Extremely	One of: • Very Slightly or Not at All • A little • Moderately • Quite a bit • Extremely	Text
panas_2	Answer to question “Distressed” Choices: • Very Slightly or Not at All • A little • Moderately • Quite a bit • Extremely	One of: • Very Slightly or Not at All • A little • Moderately • Quite a bit • Extremely	Text
panas_3	Answer to question “Excited” Choices: • Very Slightly or Not at All • A little • Moderately • Quite a bit • Extremely	One of: • Very Slightly or Not at All • A little • Moderately • Quite a bit • Extremely	Text
panas_4	Answer to question “Upset” Choices: • Very Slightly or Not at All • A little • Moderately • Quite a bit • Extremely	One of: • Very Slightly or Not at All • A little • Moderately • Quite a bit • Extremely	Text
panas_5	Answer to question “Strong” Choices: • Very Slightly or Not at All • A little • Moderately • Quite a bit • Extremely	One of: • Very Slightly or Not at All • A little • Moderately • Quite a bit • Extremely	Text
panas_6	Answer to question “Guilty” Choices: • Very Slightly or Not at All • A little • Moderately • Quite a bit • Extremely	One of: • Very Slightly or Not at All • A little • Moderately • Quite a bit • Extremely	Text
panas_7	Answer to question “Scared” Choices: • Very Slightly or Not at All • A little • Moderately • Quite a bit • Extremely	One of: • Very Slightly or Not at All • A little • Moderately • Quite a bit • Extremely	Text
panas_8	Answer to question “Hostile” Choices: • Very Slightly or Not at All • A little • Moderately • Quite a bit • Extremely	One of: • Very Slightly or Not at All • A little • Moderately • Quite a bit • Extremely	Text
panas_9	Answer to question “Enthusiastic” Choices: • Very Slightly or Not at All • A little • Moderately • Quite a bit • Extremely	One of: • Very Slightly or Not at All • A little • Moderately • Quite a bit • Extremely	Text
panas_10	Answer to question “Proud” Choices: • Very Slightly or Not at All • A little • Moderately • Quite a bit • Extremely	One of: • Very Slightly or Not at All • A little • Moderately • Quite a bit • Extremely	Text
panas_11	Answer to question “Irritable” Choices: • Very Slightly or Not at All • A little • Moderately • Quite a bit • Extremely	One of: • Very Slightly or Not at All • A little • Moderately • Quite a bit • Extremely	Text
panas_12	Answer to question “Alert” Choices: • Very Slightly or Not at All • A little • Moderately • Quite a bit • Extremely	One of: • Very Slightly or Not at All • A little • Moderately • Quite a bit • Extremely	Text
panas_13	Answer to question “Ashamed” Choices: • Very Slightly or Not at All • A little • Moderately • Quite a bit • Extremely	One of: • Very Slightly or Not at All • A little • Moderately • Quite a bit • Extremely	Text
panas_14	Answer to question “Inspired” Choices: • Very Slightly or Not at All • A little • Moderately • Quite a bit • Extremely	One of: • Very Slightly or Not at All • A little • Moderately • Quite a bit • Extremely	Text
panas_15	Answer to question “Nervous” Choices: • Very Slightly or Not at All • A little • Moderately • Quite a bit • Extremely	One of: • Very Slightly or Not at All • A little • Moderately • Quite a bit • Extremely	Text
panas_16	Answer to question “Determined” Choices: • Very Slightly or Not at All • A little • Moderately • Quite a bit • Extremely	One of: • Very Slightly or Not at All • A little • Moderately • Quite a bit • Extremely	Text
panas_17	Answer to question “Attentive” Choices: • Very Slightly or Not at All • A little • Moderately • Quite a bit • Extremely	One of: • Very Slightly or Not at All • A little • Moderately • Quite a bit • Extremely	Text
panas_18	Answer to question “Jittery” Choices: • Very Slightly or Not at All • A little • Moderately • Quite a bit • Extremely	One of: • Very Slightly or Not at All • A little • Moderately • Quite a bit • Extremely	Text
panas_19	Answer to question “Active” Choices: • Very Slightly or Not at All • A little • Moderately • Quite a bit • Extremely	One of: • Very Slightly or Not at All • A little • Moderately • Quite a bit • Extremely	Text
panas_20	Answer to question “Afraid” Choices: • Very Slightly or Not at All • A little • Moderately • Quite a bit • Extremely	One of: • Very Slightly or Not at All • A little • Moderately • Quite a bit • Extremely	Text
political	Answer to “Overall, on the following scale of political orientation (from extremely liberal to extremely conservative) where would you place yourself?Choices: • 1 - Extremely Liberal • 2 • 3 • 4 • 5 • 6 • 7 - Extremely Conservative	One of: • 1.0 • 2.0 • 3.0 • 4.0 • 5.0 • 6.0 • 7.0	Text/Number
racial_1	Answer to question “Compared to before the coronavirus, has the amount you felt you have beentreated poorly as a result of your racial, ethnic, or cultural background changed?” Choices: • It has decreased a lot • It has decreased a little • It has stayed about the same • It has increased a little • It has increased a lot	One of: • It has decreased a lot • It has decreased a little • It has stayed about the same • It has increased a little • It has increased a lot	Text
racial_2	Answer to question “I have experienced negative interactions with people in which myrace/ethnicity/culture was mentioned in connection with the coronavirus.” Choices: • Not at all true • A little bit true • Moderately true • Very True • Extremely True	One of: • Not at all true • A little bit true • Moderately true • Very True • Extremely True	Text
racial_3	Answer to question “I am worried people will blame me for the coronavirus. Choices • Not at all true • A little bit true • Moderately true • Very True • Extremely True	One of: • Not at all true • A little bit true • Moderately true • Very True • Extremely True	Text
racial_4	Answer to question “I worry that medical professionals may be biased against people of my racial/ethnic/cultural background. Choices: • Not at all true • A little bit true • Moderately true • Very True • Extremely True	One of: • Not at all true • A little bit true • Moderately true • Very True • Extremely True	Text
tam_1	Answer to “I find Personicle easy to use” Choices: • Strongly Disagree • Disagree • Slightly Disagree • Neither Agree Nor Disagree • Slightly Agree • Agree • Strongly Agree	One of: • Strongly Disagree • Disagree • Slightly Disagree • Neither Agree Nor Disagree • Slightly Agree • Agree • Strongly Agree	Text
tam_2	Answer to “Learning how to use Personicle is easy for me” Choices: • Strongly Disagree • Disagree • Slightly Disagree • Neither Agree Nor Disagree • Slightly Agree • Agree • Strongly Agree	One of: • Strongly Disagree • Disagree • Slightly Disagree • Neither Agree Nor Disagree • Slightly Agree • Agree • Strongly Agree	Text
tam_3	Answer to “It is easy to become skillful at using Personicle” Choices: • Strongly Disagree • Disagree • Slightly Disagree • Neither Agree Nor Disagree • Slightly Agree • Agree • Strongly Agree	One of: • Strongly Disagree • Disagree • Slightly Disagree • Neither Agree Nor Disagree • Slightly Agree • Agree • Strongly Agree	Text
tam_4	Answer to “Perosnicle would improve my performance” Choices: • Strongly Disagree • Disagree • Slightly Disagree • Neither Agree Nor Disagree • Slightly Agree • Agree • Strongly Agree	One of: • Strongly Disagree • Disagree • Slightly Disagree • Neither Agree Nor Disagree • Slightly Agree • Agree • Strongly Agree	Text
tam_5	Answer to “Personicle would increase productivity” Choices: • Strongly Disagree • Disagree • Slightly Disagree • Neither Agree Nor Disagree • Slightly Agree • Agree • Strongly Agree	One of: • Strongly Disagree • Disagree • Slightly Disagree • Neither Agree Nor Disagree • Slightly Agree • Agree • Strongly Agree	Text
tam_6	Answer to “Personicle could make it easier to practice” Choices: • Strongly Disagree • Disagree • Slightly Disagree • Neither Agree Nor Disagree • Slightly Agree • Agree • Strongly Agree	One of: • Strongly Disagree • Disagree • Slightly Disagree • Neither Agree Nor Disagree • Slightly Agree • Agree • Strongly Agree	Text
tam_7	Answer to “Practicing through Personicle is a good idea” Choices: • Strongly Disagree • Disagree • Slightly Disagree • Neither Agree Nor Disagree • Slightly Agree • Agree • Strongly Agree	One of: • Strongly Disagree • Disagree • Slightly Disagree • Neither Agree Nor Disagree • Slightly Agree • Agree • Strongly Agree	Text
tam_8	Answer to “Practicing through Personicle is a wise idea” Choices: • Strongly Disagree • Disagree • Slightly Disagree • Neither Agree Nor Disagree • Slightly Agree • Agree • Strongly Agree	One of: • Strongly Disagree • Disagree • Slightly Disagree • Neither Agree Nor Disagree • Slightly Agree • Agree • Strongly Agree	Text
tam_9	Answer to “I am positive toward Personicle” Choices: • Strongly Disagree • Disagree • Slightly Disagree • Neither Agree Nor Disagree • Slightly Agree • Agree • Strongly Agree	One of: • Strongly Disagree • Disagree • Slightly Disagree • Neither Agree Nor Disagree • Slightly Agree • Agree • Strongly Agree	Text
tam_10	Answer to “I intend to check announcements from Personicle frequently” Choices: • Strongly Disagree • Disagree • Slightly Disagree • Neither Agree Nor Disagree • Slightly Agree • Agree • Strongly Agree	One of: • Strongly Disagree • Disagree • Slightly Disagree • Neither Agree Nor Disagree • Slightly Agree • Agree • Strongly Agree	Text
tam_11	Answer to “I intend to be a heavy user of Personicle” Choices: • Strongly Disagree • Disagree • Slightly Disagree • Neither Agree Nor Disagree • Slightly Agree • Agree • Strongly Agree	One of: • Strongly Disagree • Disagree • Slightly Disagree • Neither Agree Nor Disagree • Slightly Agree • Agree • Strongly Agree	Text
tam_12	Answer to “I feel confident finding information in Personicle” Choices: • Strongly Disagree • Disagree • Slightly Disagree • Neither Agree Nor Disagree • Slightly Agree • Agree • Strongly Agree	One of: • Strongly Disagree • Disagree • Slightly Disagree • Neither Agree Nor Disagree • Slightly Agree • Agree • Strongly Agree	Text
tam_13	Answer to “I have the necessary skills for using Personicle” Choices: • Strongly Disagree • Disagree • Slightly Disagree • Neither Agree Nor Disagree • Slightly Agree • Agree • Strongly Agree	One of: • Strongly Disagree • Disagree • Slightly Disagree • Neither Agree Nor Disagree • Slightly Agree • Agree • Strongly Agree	Text
tam_14	Answer to “What Personicle stands for is important for me as a therapist” Choices: • Strongly Disagree • Disagree • Slightly Disagree • Neither Agree Nor Disagree • Slightly Agree • Agree • Strongly Agree	One of: • Strongly Disagree • Disagree • Slightly Disagree • Neither Agree Nor Disagree • Slightly Agree • Agree • Strongly Agree	Text
tam_15	Answer to “I like using Personicle based on the similarity of my values and society values underlying its use” Choices: • Strongly Disagree • Disagree • Slightly Disagree • Neither Agree Nor Disagree • Slightly Agree • Agree • Strongly Agree	One of: • Strongly Disagree • Disagree • Slightly Disagree • Neither Agree Nor Disagree • Slightly Agree • Agree • Strongly Agree	Text
tam_16	Answer to “In order for me to prepare for a future job, it is necessary to use Personicle” Choices: • Strongly Disagree • Disagree • Slightly Disagree • Neither Agree Nor Disagree • Slightly Agree • Agree • Strongly Agree	One of: • Strongly Disagree • Disagree • Slightly Disagree • Neither Agree Nor Disagree • Slightly Agree • Agree • Strongly Agree	Text
tam_17	Answer to “I have no difficulty accessing and using the Personicle system in my practice” Choices: • Strongly Disagree • Disagree • Slightly Disagree • Neither Agree Nor Disagree • Slightly Agree • Agree • Strongly Agree	One of: • Strongly Disagree • Disagree • Slightly Disagree • Neither Agree Nor Disagree • Slightly Agree • Agree • Strongly Agree	Text
uls_1	Answer to: “How often do you feel that you lack companionship?” Choices: • Hardly ever • Some of the time • Often	One of: • Hardly ever • Some of the time • Often	Text
uls_2	Answer to “How often do you feel left out?” Choices: • Hardly ever • Some of the time • Often	One of: • Hardly ever • Some of the time • Often	Text
uls_3	Answer to “How often do you feel isolated from others?” Choices: • Hardly ever • Some of the time • Often	One of: • Hardly ever • Some of the time • Often	Text

surveys.csv

**Table 18:** Assessment/surveys.csv.

Column name	Description	Range	Unit
start_timestamp	The timestamp that the participant has started filling out the survey.	-	Timestamp (milliseconds)
end_timestamp	The timestamp that the participant has finished filling out the survey.	-	Timestamp (milliseconds)
duration_sec	The time it took the participant to finish the survey.	-	Number (seconds)
academicevent_1	Answer to “Please rate how impactful each academic event was to you - Winter Quarter 2020.” Choices: • 1 (Not at all impactful) • 2 • 3 • 4 (Moderately Impactful) • 5 • 6 • 7 (Very Impactful)	1-7	Number
academicevent_2	Answer to “Please rate how impactful each academic event was to you - Winter Quarter 2020 Finals” Choices: • 1 (Not at all impactful) • 2 • 3 • 4 (Moderately Impactful) • 5 • 6 • 7 (Very Impactful)	1-7	Number
academicevent_3	Answer to “Please rate how impactful each academic event was to you - Spring Break” Choices: • 1 (Not at all impactful) • 2 • 3 • 4 (Moderately Impactful) • 5 • 6 • 7 (Very Impactful)	1-7	Number
academicevent_4	Answer to “Please rate how impactful each academic event was to you - Spring Quarter 2020” Choices: • 1 (Not at all impactful) • 2 • 3 • 4 (Moderately Impactful) • 5 • 6 • 7 (Very Impactful)	1-7	Number
academicevent_5	Answer to “Please rate how impactful each academic event was to you - Spring Quarter 2020 Finals” Choices: • 1 (Not at all impactful) • 2 • 3 • 4 (Moderately Impactful) • 5 • 6 • 7 (Very Impactful)	1-7	Number
academicevent_6	Answer to “Please rate how impactful each academic event was to you - Summer Session I” Choices: • 1 (Not at all impactful) • 2 • 3 • 4 (Moderately Impactful) • 5 • 6 • 7 (Very Impactful)	1-7	Number
academicevent_7	Answer to “Please rate how impactful each academic event was to you - Summer Session I Finals” Choices: • 1 (Not at all impactful) • 2 • 3 • 4 (Moderately Impactful) • 5 • 6 • 7 (Very Impactful)	1-7	Number
academicevent_8	Answer to “Please rate how impactful each academic event was to you - Summer Session II Finals” Choices: • 1 (Not at all impactful) • 2 • 3 • 4 (Moderately Impactful) • 5 • 6 • 7 (Very Impactful)	1-7	Number
academicevent_9	Answer to “Please rate how impactful each academic event was to you - Summer Session II” Choices: • 1 (Not at all impactful) • 2 • 3 • 4 (Moderately Impactful) • 5 • 6 • 7 (Very Impactful)	1-7	Number
academicevent_10	Answer to “Please rate how impactful each academic event was to you - Summer 2020 Break” Choices: • 1 (Not at all impactful) • 2 • 3 • 4 (Moderately Impactful) • 5 • 6 • 7 (Very Impactful)	1-7	Number
academicevent_11	Answer to “Please rate how impactful each academic event was to you - Fall Quarter 2020” Choices: • (Not at all impactful) • 2 • 3 • 4 (Moderately Impactful) • 5 • 6 • 7 (Very Impactful)	1-7	Number
academicevent_12	Answer to “Please rate how impactful each academic event was to you - Fall Quarter 2020 Finals” Choices: • 1 (Not at all impactful) • 2 • 3 • 4 (Moderately Impactful) • 5 • 6 • 7 (Very Impactful)	1-7	Number
academicevent_13	Answer to “Please rate how impactful each academic event was to you - Winter Break 2020-2021” Choices: • 1 (Not at all impactful) • 2 • 3 • 4 (Moderately Impactful) • 5 • 6 • 7 (Very Impactful)	1-7	Number
event_1	Answer to “Please rate how impactful each event was to you - Joe Biden wins Presidency” Choices: • 1 (Not at all impactful) • 2 • 3 • 4 (Moderately Impactful) • 5 • 6 • 7 (Very Impactful) **This field is only available upon request to protect participants’ privacy.**	1-7	Number
event_2	Answer to “Please rate how impactful each event was to you - First California Quarantine Order” Choices: • 1 (Not at all impactful) • 2 • 3 • 4 (Moderately Impactful) • 5 • 6 • 7 (Very Impactful)	1-7	Number
event_3	Answer to “Please rate how impactful each event was to you - Black Lives Matter protests” Choices: • 1 (Not at all impactful) • 2 • 3 • 4 (Moderately Impactful) • 5 • 6 • 7 (Very Impactful) **This field is only available upon request to protect participants’ privacy.**	1-7	Number
event_4	Answer to “Please rate how impactful each event was to you - Northern California Wildfires” Choices: • 1 (Not at all impactful) • 2 • 3 • 4 (Moderately Impactful) • 5 • 6 • 7 (Very Impactful)	1-7	Number
event_5	Answer to “Please rate how impactful each event was to you - Transition to Online Exams and Remote Learning” Choices: • 1 (Not at all impactful) • 2 • 3 • 4 (Moderately Impactful) • 5 • 6 • 7 (Very Impactful)	1-7	Number
event_6	Answer to “Please rate how impactful each event was to you - Original Coronavirus WHO Announcement” Choices: • 1 (Not at all impactful) • 2 • 3 • 4 (Moderately Impactful) • 5 • 6 • 7 (Very Impactful)	1-7	Number
event_7	Answer to “Please rate how impactful each event was to you - Capitol Riots” Choices: • 1 (Not at all impactful) • 2 • 3 • 4 (Moderately Impactful) • 5 • 6 • 7 (Very Impactful) **This field is only available upon request to protect participants’ privacy.**	1-7	Number
event_8	Answer to “Please rate how impactful each event was to you - Death of George Floyd” Choices: • 1 (Not at all impactful) • 2 • 3 • 4 (Moderately Impactful) • 5 • 6 • 7 (Very Impactful) **This field is only available upon request to protect participants’ privacy.**	1-7	Number
event_9	Answer to “Please rate how impactful each event was to you - Death of Ruth Bader-Ginsburg” Choices: • 1 (Not at all impactful) • 2 • 3 • 4 (Moderately Impactful) • 5 • 6 • 7 (Very Impactful) **This field is only available upon request to protect participants’ privacy.**	1-7	Number
event_10	Answer to “Please rate how impactful each event was to you - Donald Trump Impeached for the First Time” Choices: • 1 (Not at all impactful) • 2 • 3 • 4 (Moderately Impactful) • 5 • 6 • 7 (Very Impactful) **This field is only available upon request to protect participants’ privacy.**	1-7	Number
event_11	Answer to “Please rate how impactful each event was to you - Death of Breonna Taylor” Choices: • 1 (Not at all impactful) • 2 • 3 • 4 (Moderately Impactful) • 5 • 6 • 7 (Very Impactful) **This field is only available upon request to protect participants’ privacy.**	1-7	Number
event_12	Answer to “Please rate how impactful each event was to you - Impact of COVID on Student Financial Aid” Choices: • 1 (Not at all impactful) • 2 • 3 • 4 (Moderately Impactful) • 5 • 6 • 7 (Very Impactful)	1-7	Number
event_13	Answer to “Please rate how impactful each event was to you - First COVID-19 Vaccine Rollout” Choices: • 1 (Not at all impactful) • 2 • 3 • 4 (Moderately Impactful) • 5 • 6 • 7 (Very Impactful)	1-7	Number
event_14	Answer to “Please rate how impactful each event was to you - Kobe Bryant Helicopter Crash/Death” Choices: • 1 (Not at all impactful) • 2 • 3 • 4 (Moderately Impactful) • 5 • 6 • 7 (Very Impactful) **This field is only available upon request to protect participants’ privacy.**	1-7	Number
event_15	Answer to “Please rate how impactful each event was to you - Supreme Court Rules LGBTQ Employees are Protected by Civil Rights Employment Statutes” Choices: • 1 (Not at all impactful) • 2 • 3 • 4 (Moderately Impactful) • 5 • 6 • 7 (Very Impactful) **This field is only available upon request to protect participants’ privacy.**	1-7	Number
event_16	Answer to “Please rate how impactful each event was to you - Trump Impeached for Second Time” Choices: • 1 (Not at all impactful) • 2 • 3 • 4 (Moderately Impactful) • 5 • 6 • 7 (Very Impactful) **This field is only available upon request to protect participants’ privacy.**	1-7	Number
event_17	Answer to “Please rate how impactful each event was to you - First stimulus check” Choices: • 1 (Not at all impactful) • 2 • 3 • 4 (Moderately Impactful) • 5 • 6 • 7 (Very Impactful)	1-7	Number
event_18	Answer to “Please rate how impactful each event was to you - Jacob Blake Shooting” Choices: • 1 (Not at all impactful) • 2 • 3 • 4 (Moderately Impactful) • 5 • 6 • 7 (Very Impactful) **This field is only available upon request to protect participants’ privacy.**	1-7	Number
event_19	Answer to “Please rate how impactful each event was to you - President Trump Tests Positive for COVID” Choices: • 1 (Not at all impactful) • 2 • 3 • 4 (Moderately Impactful) • 5 • 6 • 7 (Very Impactful) **This field is only available upon request to protect participants’ privacy.**	1-7	Number
event_20	Answer to “Please rate how impactful each event was to you - Supreme Court Rules Against Ending DACA” Choices: • 1 (Not at all impactful) • 2 • 3 • 4 (Moderately Impactful) • 5 • 6 • 7 (Very Impactful) **This field is only available upon request to protect participants’ privacy.**	1-7	Number
gear_1	Answer to “What features of the Gear Sport watch did you like?” **This field is only available upon request to protect participants’ privacy.**	–	Text
gear_2	Answer to “What features did you find irritating? What features would need to be changed or added in order for it not to be irritating?” **This field is only available upon request to protect participants’ privacy.**	–	Text
gear_3	Answer to “Did you find the Gear Sport watch helpful? Why or why not?” **This field is only available upon request to protect participants’ privacy.**	–	Text
gear_4	Answer to “How do you feel about how the data/information is presented back to you?” **This field is only available upon request to protect participants’ privacy.**	–	Text
gear_5	Answer to “What questions do you have about your data that the Gear Sport watch doesn't answer?” **This field is only available upon request to protect participants’ privacy.**	–	Text
gear_6	Answer to “What do you think is missing from the Gear Sport watch, if anything?” **This field is only available upon request to protect participants’ privacy.**	–	Text
gear_7	Answer to “Do you believe the measurements displayed on the application accurately reflect reality? If not, please elaborate on these inaccuracies.” **This field is only available upon request to protect participants’ privacy.**	–	Text
gear_8_1	Answer to “How likely would you be to adopt the watch if you had the option to use it? - 1” **This field is only available upon request to protect participants’ privacy.**	–	Text
gear_9	Answer to “What features would need to be changed or added in order for you to adopt it, if any?” **This field is only available upon request to protect participants’ privacy.**	–	Text
gear_10	Answer to “If a friend who was interested in the Gear Sport watch came and asked you about it, what would you tell them?” **This field is only available upon request to protect participants’ privacy.**	–	Text
general_1	Answer to “What features of the overall study did you like?” **This field is only available upon request to protect participants’ privacy.**	–	Text
general_2	Answer to “What aspects did you find irritating? Are there things/features you would want to change? What would this look like?” **This field is only available upon request to protect participants’ privacy.**	–	Text
general_3	Answer to “Do you believe the Personicle system as a whole (daily emotion surveys, smart devices) could be used as a successful intervention to promote mental health? Please describe your reasoning.” **This field is only available upon request to protect participants’ privacy.**	–	Text
general_4	Answer to “Since joining the study have you noticed the Personicle system as a whole to have an impact on your life? If so, please describe in detail.” **This field is only available upon request to protect participants’ privacy.**	–	Text
general_5	Answer to “Did you ever/ do you currently have any concerns regarding the privacy and security of the data being collected by any of the applications? If so, please describe in detail.” **This field is only available upon request to protect participants’ privacy.**	–	Text
international	Answer to “Are you an international student?” Choices: • Yes • No **This field is only available upon request to protect participants’ privacy.**	One of: • Yes • No	Text
oura_1	Answer to “What features of the Oura Ring did you like?” **This field is only available upon request to protect participants’ privacy.**	–	Text
oura_2	Answer to “What features did you find irritating? What features would need to be changed or added in order for it not to be irritating?” **This field is only available upon request to protect participants’ privacy.**	–	Text
oura_3	Answer to “Did you find the Oura Ring helpful? Why or why not?” **This field is only available upon request to protect participants’ privacy.**	–	Text
oura_4	Answer to “How do you feel about how the data/information is presented back to you?” **This field is only available upon request to protect participants’ privacy.**	–	Text
oura_5	Answer to “What questions do you have about your data that the Oura Ring doesn't answer?” **This field is only available upon request to protect participants’ privacy.**	–	Text
oura_6	Answer to “What do you think is missing from the Oura Ring, if anything?” **This field is only available upon request to protect participants’ privacy.**	–	Text
oura_7	Answer to “Do you believe the measurements displayed on the application accurately reflect reality? If not, please elaborate on these inaccuracies.” **This field is only available upon request to protect participants’ privacy.**	–	Text
oura_8_1	Answer to “How likely would you be to adopt the ring if you had the option to use it? - 1” **This field is only available upon request to protect participants’ privacy.**	–	Text
oura_9	Answer to “What features would need to be changed or added in order for you to adopt it, if any?” **This field is only available upon request to protect participants’ privacy.**	–	Text
oura_10	Answer to “If a friend who was interested in the Oura Ring came and asked you about it, what would you tell them?” **This field is only available upon request to protect participants’ privacy.**	–	Text
personicle_app_5	Answer to “What questions do you have about your data that the Personicle app doesn't answer?” **This field is only available upon request to protect participants’ privacy.**	–	Text
personicle_app_6	Answer to “What do you think is missing from Personicle, if anything?” **This field is only available upon request to protect participants’ privacy.**	–	Text
personicle_app_7	Answer to “Do you believe the measurements displayed on the application accurately reflect reality? If not, please elaborate on these inaccuracies.” **This field is only available upon request to protect participants’ privacy.**	–	Text
personicle_app_8_1	Answer to “How likely would you be to adopt the application if you had the option to use it? - 1” **This field is only available upon request to protect participants’ privacy.**	–	Text
personicle_app_9	Answer to “What features would need to be changed or added in order for you to adopt it, if any?” **This field is only available upon request to protect participants’ privacy.**	–	Text
personicle_app_10	Answer to “If a friend who was interested in Personicle came and asked you about it, what would you tell them?” **This field is only available upon request to protect participants’ privacy.**	–	Text
resources_1	Answer to “Have you utilized any of the following resources since the beginning of your enrollment in the study? - Selected Choice” Choices: • Psychotherapy • Psychotropic Medication (e.g. SSRI) • Support Group • Religious/spiritual supports (e.g., retreats, bible study, group meditation, etc.) • Other **This field is only available upon request to protect participants’ privacy.**	One of: • Psychotherapy • Psychotropic Medication (e.g. SSRI) • Support Group • Religious/spiritual supports (e.g., retreats, bible study, group meditation, etc.) • Other	Text
resources_1_5_text	Answer to “Have you utilized any of the following resources since the beginning of your enrollment in the study? - Selected “Other”” **This field is only available upon request to protect participants’ privacy.**	–	Text
resources_2	Answer to “Have you had as much access to resources to support your mental health as you would like during your enrollment in the study?” Choices: • Yes • No • N/A	One of: • Yes • No • N/A	Text
resources_3	Answer to “Have you utilized UCI's Wellness, Health, & Counseling Services?” Choices: Yes No **This field is only available upon request to protect participants’ privacy.**	One of: • Yes • No	Text
vote	Answer to “Did you vote in the 2020 United States Presidential Election?” Choices: • Yes • No	One of: • Yes • No	Text

**Table 19:** Personicle/personicle.csv.

**Column name**	**Description**	**Range**	**Type of variable**
timestamp	Starting Epoch timestamp of the data segment.	-	Timestamp
activity_level	Average score of the physical activity set $C\;=\;\sum_{i=0}^{4}\frac{i\;\times {\left(PhysicalActivity\right)}_{i}}{k}$ We have 5 types of activities and they are scored from 0 to 4. Then activity level can be calculated based on the average of the frequency of each activity in five minute time frames. [6]	-	Number
activity_name	Name of the current activity.	-	Text
sub_activity_name	Name of the current Sub-activity.	-	Text
activity_type	Activity type extracted from Google activity detector. Valid values: • IN_VEHICLE • ON_BICYCLE • ON_FOOT • RUNNING • STILL • TILTING • UNKNOWN • WALKING	-	Text
activity_previous_event	The name of the previous event which is the previous segment activity_name of the same day.	-	Text
venue_name	The name of the venue the user is currently in which is extracted from google location service. **This field is only available upon request to protect participants’ privacy.**	-	Text
venue_name_arrival	The name of the destination venue extracted from google location service. **This field is only available upon request to protect participants’ privacy.**	-	Text
venue_name_departure	The name of the departure venue extracted from google location service. **This field is only available upon request to protect participants’ privacy.**	-	Text
venue_type	The type of the venu extracted from Google location service.	-	Text
time_band	List of timebands the current segment is in. • 0: 00:00 - 03:59 • 1: 04:00 - 07:59 • 2: 08:00 - 11:59 • 3: 12:00 - 15:59 • 4: 16:00 - 19:59 • 5: 20:00 - 23:59 example: [1,2]	-	Text
major_activity	The name of the major activity done in the current segment based on extracted activity type from Google activity detector. Valid values: • IN_VEHICLE • ON_BICYCLE • ON_FOOT • RUNNING • STILL • TILTING • UNKNOWN • WALKING	-	Text
activity_duration	Indicates the qualitative activity duration: • very short • short • long • very long	-	Text
time_window	The 5 minutes-based indexing. Each 5 minutes is 1 index and one day has 288 5 minutes.	-	Text
step_count	Aggregated step count from the start of the day till the current segment timestamp.	-	Number

personicle.csv.

#### 2.3.5 Personicle

The data is collected using Personicle mobile application [6]. It records life logs of participants and converts them into event segments including location and activity information of participants. Segments indicate the 5 minutes interval of event changes. If two consecutive events have the same label, they will be stored as one with the start time of the earliest happening event. As soon as the segment label changes, a new event segment is created. The data collected via Personicle mobile application is presentated in Table 19.

### 2.4 Code

There is going to be a “ifh_affect.py” file alongside the dataset folders. This file does not contain any data but it can facilitate loading and transforming the data in Python.

This file provides two classes DataLoader and DataTransform. The DataLoader class can take the address of the dataset root or zipfile and load different modalities for each participant or for multiple of them. The DataTransform function provides functions for converting the timestamps to human readable times.

## 3 Experimental Design, Materials and Methods

### 3.1 Experiment design and setup

This study was designed to create a longitudinal dataset of physiological and emotional assessments for emerging adults. This dataset can be used for studying affect and correlations of mental health, affect, physiology, sleep, and activity of emerging adults.

However, as COVID-19 pandemic and lockdown started during the study, the purpose of this study was shifted toward studying the effects of the lockdown on participants' life and mental health. To adapt to the new conditions, the study design, recruitment materials, and questionnaires were updated as the study was ongoing.

This study collected objective ubiquitous data from wearable devices and life-logger apps and combined them with subjective ecological momentary assessments (EMAs) and surveys to create a spectrum of physiological and mental profile for each participant over time. To achieve this objective, this study was required to collect different modalities of data. First, the participants needed to be objectively monitored for their sleep and activity. While many wearable devices can provide accurate measurements on sleep and activity, collecting raw data can be useful in extracting additional features and creating opportunities for future research. Second, the participants' mood and mental state needed to be monitored by subjectively asking them about their mood and mental state.

In order to collect this dataset, it was necessary to build an Internet platform that can ubiquitously monitor the participants over time, and provide tools for the study coordinator to monitor the progress and data collection remotely. To this end, ZotCare [7] was utilised. ZotCare is an online mHealth service that can be used to collect, store, and analyse the data on different levels of technology stack. Services used in this study include the collection service for collecting objective data from participants' wearable devices and collecting subjective EMAs through mobile app. The collected data then was processed by ZotCare's cloud server and stored in a database. Besides that, ZotCare's dashboard was utilised in this study to monitor the collected data and ensure participants' collaboration in data collection.

To collect the subjective EMAs, ZotCare's frontend app was customised in this study and was published as Personicle Questions in Google Play. In this application participants would answer daily EMA questionnaires and weekly ones. Both EMAs contain questionnaires that focus on participants’ emotions and their worries about their health in regards to COVID-19. The weekly EMAs additionally contains participants’ written responses about the highs and lows of the previous week. The emotion questions stem from an established measurement, the Positive and Negative Affect Scale (PANAS) [6], while others are brief self-developed, single-item questions that help get an understanding of participants’ overall emotions and worries as a result of the largely-impactful event at the time, the COVID-19 pandemic. The participants could answer the daily questionnaire from 7:50 PM to 3:00 AM PST time. Every participant was notified at 8PM every night to respond to this questionnaire. If the participant did not respond to the EMA, two follow up reminders would be sent to them at 10PM and 11:59 PM. The weekly EMA was open for participants from 7:50 AM to 8 PM PST time every Sunday with the first reminder at 8 AM and two follow up reminders at 10 AM and noon in case they failed to respond to the EMA before the reminder.

In order to collect raw objective data of the study, ZotCare Tizen wearable app was used to collect the data directly from the participant's watch. This app was set to collect raw data from sensors such as ppg, accelerometer, gyroscope with frequency of 20Hz, and pressure with frequency of 10Hz every 2 hours for 12 minutes. Besides the raw sensors, the samsung watch also collected data for pedometer and awake times. The pedometer data is supposed to contain step counts and more details of the participants' stepping pace. The frequency of pedometer data is random since it depends on the frequency of the steps taken and its detection by the watch. The awake times data shows the times that the watch detects that the participant is awake.

In this experiment, Oura ring was used to monitor participants' activity and sleep. Oura is one of the most accurate commercially available wearable devices for sleep monitoring and its battery life is higher than smartwatches. The data collected by Oura is stored in Oura's servers. ZotCare can use Open Authentication to collect these data from Oura and make them available to researchers on daily bases.

The Personicle Android mobile application was used to collect participants' data, which collected and processed the Google location, Google place, mobile device-specific measures like Calendar, and Ambient Light to identify points of interest and predict the activities performed by the user on a daily basis. Datapoints Personicle, as a mobile application, collected the phone data and sent it to the server for further processing, which, in turn, created the following data points: **Lifelog**: This was the raw data collected on every 5-minute interval from the phone. **Point of interest** (POI): This was the derived location/place of a user based on the state of the user (still/motion), Google location, and place API. Home and Work locations were also set as POI after collecting the data for 2 days from the date of app installation. **Segment**: Segments, in general, were the aggregated/filtered results from lifelog and POI between the occurrences of events. Changes in the state (still/motion) of a person or events like shopping or sleep were considered as events. We are providing access to Segments in this dataset.

The last data collection modality was subjective assessments. These assessments were collected upon participant's enrollment, their exit, and every month of their participation in the study. Also another assessment was collected from participants about the events that have happened during their enrollment in the study and their effect on their mental state and emotions.These assessments were developed using Qualtrics software. Each assessment was a combination of different standard and self-developed questionnaires that is represented in the Table 20.

**Table 20:** Sources of all the assessment questionnaires.

Measure	Description	Source
Technology Acceptance Model Measurement (Modified)	Modified to assess a user's acceptance of the Personicle application/device. The constructs include perceived ease of use, perceived usefulness, attitude, behavioural intention, self-efficacy, subjective norm, and system accessibility.	[9]
Acceptability of Intervention Measure (AIM-Modified), Intervention Appropriateness Measure (IAM- Modified), and Feasibility of Intervention Measure (FIM-Modified)	Assesses the implementation outcomes of the Personicle devices and application in terms of acceptability, appropriateness and feasibility.	[10]
Beck Depression Inventory II (BDI-II)	Assesses an individual's depression severity ranging from mild to severe.	[11]
Positive and Negative Affect Schedule (PANAS-SF)	Measures an individual's positive and negative emotions.	[8]
Brief Symptom Inventory (BSI)	Evaluates current or past level of symptomatology, intensity of symptoms, and number of reported symptoms. The questionnaire covers nine dimensions of symptoms including somatization, obsession-compulsion, interpersonal sensitivity, depression, anxiety, hostility, phobic anxiety, paranoia, and psychosis.	[12]
Experiences in Close Relationships Scale Short Form (ECR-S)	Assesses attachment avoidance and attachment anxiety.	[13]
UCLA Three-Item Loneliness Scale (ULS)	Assesses loneliness.	[14]
Multidimensional Scale of Perceived Social Support (MSPSS)	Assesses social support from family, friends, and significant other.	[15]
Investment Model Scale (IMS)	Measures four constructs (commitment level, satisfaction level, quality of alternatives, an investment size).	[16]
Inclusion of Other in the Self Scale (IOS-Partner)	Measures how close the respondent feels with another person (romantic partner).	[17]
Conflict Management Scale (CMS)	Assesses conflict management in romantic relationships.	[18]
GAD-7	A brief scale that is used to help identify cases of Generalized Anxiety Disorder.	[19]
Social Isolation	Assesses social isolation in relation to the COVID-19 pandemic.	*Manuscript in progress*
Racial Profiling/Bias	Measures how the respondent interprets interactions with others based on race after the COVID-19 pandemic.	*Manuscript in progress*
Health Exposure	Measures the degree of exposure to the coronavirus.	*Manuscript in progress*
Health Mindset	Measures an individual's opinion on the effect size of changes in one's physical health.	*Manuscript in progress*
Health Behavioral Changes & Health Impact	Assesses behavioral changes relating to health in conjunction to the COVID-19 pandemic.	*Manuscript in progress*
Health Anxiety	Measures how stressed and/or anxious an individual is over their health regarding the COVID-19 pandemic.	*Manuscript in progress*
Health Perceived Control	Measures an individual's perceived control over their health in relation to the COVID-19 pandemic.	*Manuscript in progress*
Health Perceived Threat	Assesses the degree that the respondent believes the Coronavirus has or will impact their health.	*Manuscript in progress*
Belief in Conspiracy Theories	A brief scale assessing an individual's belief in conspiracy theories in relation to the COVID-19 pandemic.	*Manuscript in progress*
Political Orientation	A single question assessing the degree of political orientation from liberal to conservative.	*Manuscript in progress*
Emotion Regulation	Measures how respondents manage emotions in relation to the COVID-19 pandemic and the events surrounding it.	*Manuscript in progress*
Brief COPE	Measures efforts of managing stressful events in relation to the COVID-19 pandemic.	[20]

### 3.2 Recruitment and Enrollment

Participants were recruited through UCI faculty and through posted flyers around UCI campus. Participants were between the ages of 18-22, enrolled at the time as UCI students, were able to fluently speak and write in English, used an Android operating system of 6.0 or higher on their primary phone (to be compatible with wearable devices), was not a parent, was not married, did not come back to school after more than three years, and was not diagnosed with or met criterias for depression. These criterias were screened for via a phone call after participants expressed interest in participating via email.

If interested participants were eligible to participate, they were then scheduled for an enrollment session. Participants visited Dr. Borelli's THRIVE lab at University of California, Irvine to complete the pre-assessment. During this enrollment session, participants completed a battery in which they were asked specific questions that re-confirmed eligibility for the study. Additionally, we collected select demographic information from them (i.e., age, year in school, gender, ethnicity). The research assistant provided information about the study and reviewed the consent form. Once the participant consented, the participant then completed psychological assessments (e.g., BDI-II, PANAS, BSI). If during the pre-assessment, the participant happened to score high on the BDI-II (i.e., a score that indicates moderate depression) or if the participant indicated suicidality, the PI, Dr. Borelli who is a liscensed clinical psychologist, contacted the participant to perform additional psychological assessments on suicide, depression, and social support screening before deciding if the participant can continue in the study or should be withdrawn. In case the participant needed additional assistance, health referrals and resources were provided to them and they were compensated in a prorated way.

If the participants were selected for the study, they had to go through an enrollment session. During the enrollment session, the research team helped set up the wearable smartwatch and smartring, download all necessary applications onto the participants’ phone (Personicle, Personicle Questions, Oura, Galaxy Wearable), and review device care and study expectations. After the session, participants would then receive an email with $30 of compensation in the form of an Amazon gift-code.

The majority of the participants were recruited during the COVID-19 pandemic. To address the social distancing orders, we modified our procedures so that the enrollment process could be completed 100% remotely. Participants would be asked to measure their ring at home (this was done using a measuring tape) to get an estimation of their ring size for the Oura ring. Participants were then mailed a shipping package, which contained the monitoring devices (watch and Oura ring). In this package, they were also given a prepaid postage stamp and an address label for them to use when returning their devices. Participants were given instructions for how to use the devices during a remote pre-assessment visit with a research member, which was accomplished via Zoom. During this remote session, the research team would guide the participants through the same procedures as an in-person visit and participants were compensated the same amount after the session.

### 3.3 Data collection

Participation was originally set for 3 months, but participants had the option to continue for an additional 3 months afterwards, up to 3 more times for a maximum of 12 months of total participation. Participants completed a followup assessment at the end of the initial 3-month study period, and completed additional follow ups after each 3-month period if they opted in. Our research team would monitor for incoming data daily and would send reminders to participants to complete surveys if 2-3 were incomplete in a row. If watch or ring data was not submitted regularly, our research team would email the participant a reminder to wear the device and would follow-up with troubleshooting methods if necessary. Participants were compensated $15 per week via email in the form of an Amazon gift-code.

### 3.4 Exit

At the end of the 12 week period, participants were emailed a Qualtrics link with their ID# for them to complete their exit assessment which took approximately 30 minutes. The exit assessment consisted of the same questions as the baseline assessment in addition to event-mining questions that asked participants to rate how large events impacted their life (i.e.,the impact of the first COVID-19 vaccine rollout). Participants were then given instructions to delete all the phone applications that were downloaded during the start of the study (Personicle, Personicle Questions, Oura, Galaxy Wearable).

All participants who were enrolled in the study between January 2020 and March 2020 were invited to an extension of the current study. At the end of the initial 12-week period, participants completed their exit survey (30 minutes). After completion of the survey, the research coordinator would then review the participant's scores on the BDI-II and follow the same protocol for screening and professional assistance as during the enrollment session. Participants were then given the opportunity to electronically sign a new consent form that asked them to consent to be involved in a continuation of the study (this occurred via DocuSign). If participants agreed to participate in the study continuation, they then continued wearing the devices and completing the surveys for an extended period of 8 weeks. These 8 weeks would have the same on-going data collection, daily surveys, and weekly surveys. At the end of the extended 8 weeks, participants were provided with a Qualtrics link for their second and final exit survey. Participants were then given instructions to delete all the phone applications that were downloaded during the start of the study (Personicle, Personicle Questions, Oura, Galaxy Wearable) and how to delete the watch's MAC address off of their OIT account. They were then asked to return the devices and chargers by using the packaging, postage stamp, and address label that was provided to them when they first received the devices. Participants were then compensated $50 via email in the form of Amazon gift-codes. Participants who decide to continue participation into the study's extension would receive an additional $50 after the second exit assessment was completed.

## Limitations

IoT device missing data: Working with wearable devices in longitudinal study can cause many missing data. Data collection might occasionally be overlooked due to unintentional oversights from participants or unforeseen technical issues.. Participants often forget to wear their devices, charge them, keep their bluetooth and Internet connection on, and also sometimes they might restart the devices or log out of their accounts by mistake and cause data loss. Besides that, we faced some technical challenges collecting data from participants. Modern operating systems such as Android and Tizen shut down applications that users do not interact with. This feature affected our Tizen application and the Personicle application since the apps were only background monitoring and users did not interact with. Another type of technical issues came from our services being down from time to time since the services used in this study were in the development stage.

## Ethics Statement

This study was approved by the institutional review board at the University of California, Irvine (approval number: 2019-5153).

## CRediT authorship contribution statement

**Sina Labbaf:** Methodology, Software, Formal analysis, Data curation, Writing – original draft. **Mahyar Abbasian:** Writing – original draft. **Brenda Nguyen:** Data curation, Writing – original draft. **Matthew Lucero:** Writing – original draft. **Maryam Sabah Ahmed:** Writing – original draft. **Asal Yunusova:** Project administration. **Alexander Rivera:** Project administration. **Ramesh Jain:** Supervision. **Jessica L. Borelli:** Supervision, Writing – review & editing. **Nikil Dutt:** Supervision, Writing – review & editing. **Amir M. Rahmani:** Supervision, Writing – review & editing.

## Data Availability

Physiological and Emotional Assessment of College Students using Wearable and Mobile Devices during the 2020 COVID-19 Lockdown: An Intensive, Longitudinal Dataset (Original data) (Dryad)
